# Effects of Vendor and Genetic Background on the Composition of the Fecal Microbiota of Inbred Mice

**DOI:** 10.1371/journal.pone.0116704

**Published:** 2015-02-12

**Authors:** Aaron C. Ericsson, J. Wade Davis, William Spollen, Nathan Bivens, Scott Givan, Catherine E. Hagan, Mark McIntosh, Craig L. Franklin

**Affiliations:** 1 Department of Veterinary Pathobiology, University of Missouri, Columbia, Missouri, United States of America; 2 Mutant Mouse Resource and Research Center, University of Missouri, Columbia, Missouri, United States of America; 3 University of Missouri Metagenomics Center, University of Missouri, Columbia, Missouri, United States of America; 4 Department of Biostatistics, University of Missouri, Columbia, Missouri, United States of America; 5 Informatics Research Core Facility, University of Missouri, Columbia, Missouri, United States of America; 6 DNA Core Facility, University of Missouri, Columbia, Missouri, United States of America; 7 Department of Molecular Microbiology and Immunology, University of Missouri, Columbia, Missouri, United States of America; Charité, Campus Benjamin Franklin, GERMANY

## Abstract

The commensal gut microbiota has been implicated as a determinant in several human diseases and conditions. There is mounting evidence that the gut microbiota of laboratory mice (*Mus musculus*) similarly modulates the phenotype of mouse models used to study human disease and development. While differing model phenotypes have been reported using mice purchased from different vendors, the composition and uniformity of the fecal microbiota in mice of various genetic backgrounds from different vendors is unclear. Using culture-independent methods and robust statistical analysis, we demonstrate significant differences in the richness and diversity of fecal microbial populations in mice purchased from two large commercial vendors. Moreover, the abundance of many operational taxonomic units, often identified to the species level, as well as several higher taxa, differed in vendor- and strain-dependent manners. Such differences were evident in the fecal microbiota of weanling mice and persisted throughout the study, to twenty-four weeks of age. These data provide the first in-depth analysis of the developmental trajectory of the fecal microbiota in mice from different vendors, and a starting point from which researchers may be able to refine animal models affected by differences in the gut microbiota and thus possibly reduce the number of animals required to perform studies with sufficient statistical power.

## Introduction

The commensal gut microbiota comprises hundreds of distinct microbial species which outnumber host somatic cells by an order of magnitude. Research on the role of the microbiota in host health and disease has emerged as a rapidly expanding area of discovery, largely due to the development of culture-independent molecular techniques such as next-generation sequencing (NGS) [1]. Using these methodologies, associations have been made between a broad range of diseases and conditions and the human fecal microbiota (FM) [2]. Owing to the relative novelty of these techniques however, the vast majority of these associations are purely correlative [3]. Additionally, associations between the microbiota and human conditions are difficult to interpret in light of the numerous variables affecting an outbred population living in diverse environmental conditions. Thus, animal models, in which genetics and the external environment can be controlled and the FM can be manipulated as an independent variable, are needed to demonstrate causative relationships between the FM and associated diseases and conditions. An essential corollary to this line of inquiry is the uniformity of intestinal microbial populations in the animals used to model human disease, and whether any inherent differences impact those models. Considering recent calls for measures to enhance the reproducibility of preclinical biomedical research [4, 5], variability in the FM of research animals represents a potential confounding factor and necessary consideration for users of animal models.

There has long been anecdotal evidence of FM-mediated changes or losses of model phenotypes, often occurring in conjunction with a change in husbandry, institution, or animal source. Several reports now provide empirical evidence that the FM can significantly impact model phenotypes [6–10]. A better understanding of which microbes affect animal models will allow for refinement of those models and subsequent reduction of animal numbers needed, as well as mechanistic clues related to the diseases under study. A logical first step toward these goals is thorough characterization of the “normal” FM of inbred laboratory mice and the impact of commonly encountered variables on its composition. The host genetic background has a consistent and reproducible impact on the composition of the FM. Co-housed mice of differing genetic backgrounds tend to maintain the inherent interstrain variation of the FM despite the practice of coprophagy [11]. Moreover, host genetic background is a greater determinant of FM composition than is host sex [12]. Similarly, other groups have demonstrated that the FM of laboratory mice differs based on strain and commercial source of the mice [13, 14] although the nature of those differences remains undetermined. That said, the microbiota of mice obtained from a common source but housed in different facilities from an early age will drift to a facility-specific composition [14], reflecting the influence of external factors such as diet and barrier status.

To our knowledge, no NGS-based studies have specifically examined vendor- and strain-dependent effects on the composition of the FM of laboratory mice. Thus, we performed Illumina-based sequencing of the hypervariable V4 region of the bacterial 16S rDNA gene using DNA extracted from the feces of multiple inbred strains of mouse, each purchased from two vendors. Additionally, two orders of all strains of mouse from each vendor, placed one week apart, were analyzed. Samples were collected and analyzed at 3.5 weeks (3.5w), 7.5w, 10.5w, and 24w of age from the same cohort of mice. Robust statistical methods were used to characterize the FM of laboratory mice, and to formally test for vendor- and strain-dependent differences in the abundance of taxa detected via 16S rRNA amplicon sequencing.

## Materials and Methods

### Ethics statement

All studies were performed in accordance with the recommendations put forth in the Guide for the Care and Use of Laboratory Animals and were approved by the University of Missouri Institutional Animal Care and Use Committee (MU IACUC protocol #7749).

### Mice

Female A/J, BALB/cJ, and C57BL/6J (Jackson Laboratory, Sacramento, CA and Bar Harbor, ME); and A/JOlaHsd, BALB/cAnNHsd, and C57BL/6NHsd (Harlan Sprague Dawley, Indianapolis, IN) mice (*n* = 16) were purchased as 3 week old weanlings and housed throughout the duration of the present studies under barrier conditions in microisolator cages with compressed pelleted paper bedding and nestlets, on ventilated racks (Thoren, Hazleton, PA) with *ad libitum* access to irradiated chow (LabDiet 5058, LabDiet, St. Louis, MO) and acidified, autoclaved water, under a 14:10 light/dark cycle. Water was acidified using an automated bottle filler (model 9WEF, Tecniplast, Buguggiate, Italy) designed to titrate municipal water with sulfuric acid to a target pH of 2.5 (range 2.3 to 2.7). Mice were determined to be free of all overt and opportunist bacterial pathogens including *Bordetella bronchiseptica*, cilia-associated respiratory (CAR) bacillus, *Citrobacter rodentium*, *Clostridium piliforme*, *Corynebacterium bovis*, *Corynebacterium kutscheri*, *Helicobacter* spp., *Mycoplasma* spp., *Pasteurella pneumotropica*, *Pneumocystis carinii*, *Salmonella* spp., *Streptobacillus moniliformis*, *Streptococcus pneumoniae*; adventitious viruses including H1, Hantaan, KRV, LCMV, MAD1, MNV, PVM, RCV/SDAV, REO3, RMV, RPV, RTV, and Sendai viruses; intestinal protozoa including *Spironucleus muris*, *Giardia muris*, *Entamoeba muris*, trichomonads, and other large intestinal flagellates and amoebae; intestinal parasites including pinworms and tapeworms; and external parasites including all species of lice and mites, via quarterly sentinel testing performed by IDEXX BioResearch (Columbia, MO). Mice from Jackson and Harlan were fed LabDiet 5K0Q or Teklad Global 2018S respectively prior to shipping. The same cohort of mice, purchased during April of 2013, provided samples at each time point. Mice were allowed to acclimate for a period of three days prior to the initial sample collection. Each group was purchased as two separate orders (*n* = 8 mice/strain/vendor for a total of 96 mice) one week apart with no additional instructions for the vendor. All strains of mice were supplied by the Bar Harbor facility in the first of two separate orders to The Jackson Laboratory, and from the Sacramento facility in the second order. All mice from Harlan Laboratories originated from the same facility.

### Sample collection and DNA extraction

Two freshly evacuated fecal pellets were collected at the same time of day (between 6 and 8 a.m.) from each mouse at every time point, i.e., at 3.5 weeks (weaning; 3.5w), 7.5w, 10.5w, and 24w of age, directly into a 2 mL round-bottom tube containing lysis buffer adapted from Yu et al. [15] and a 0.5 cm diameter stainless steel bead. Following mechanical disruption using a TissueLyser II (Qiagen, Venlo, Netherlands), tubes were incubated at 70°C for 20 minutes with periodic vortexing. Samples were then centrifuged at 5000×g for five minutes at room temperature, and the supernatant transferred to a clean 1.5 mL Eppendorf tube. Two hundred μL of 10 mM ammonium acetate was added to lysates, mixed thoroughly, incubated on ice for five minutes, and then centrifuged as above. Supernatant was then mixed thoroughly with one volume of chilled isopropanol and incubated on ice for 30 minutes. Samples were then centrifuged at 16000×g for 15 minutes at 4°C. The supernatant was aspirated and discarded, and the DNA pellet washed several times with 70% ethanol and resuspended in 150 μL of Tris-EDTA. 15 μL of proteinase-K and 200 μL of Buffer AL (DNeasy Blood and Tissue kit, Qiagen) were added and samples were incubated at 70°C for 10 minutes. 200 μL of 100% ethanol was added and the contents of each tube were transferred to a spin column from the DNeasy kit. DNA was then purified according to the manufacturer’s instructions and eluted in 200 μL of EB buffer (Qiagen). Purity of DNA was assessed via spectrophotometry (Nanodrop, Thermo Fisher Scientific, Waltham, MA); yield was determined via fluorometry (Qubit, Life Technologies, Carlsbad, CA) using quant-iT BR dsDNA reagent kit (Invitrogen, Carlsbad, CA).

### 16S rRNA library preparation and sequencing

Extracted fecal DNA was processed at the University of Missouri DNA Core Facility. Bacterial 16S rDNA amplicons were constructed via amplification of the V4 hypervariable region of the 16s rDNA gene with universal primers (U515F/806R) previously developed against the V4 region, flanked by Illumina standard adapter sequences [16, 17]. Oligonucleotide sequences are available at proBase [18]. A single forward primer and reverse primers with a unique 12-base index were used in all reactions. PCR reactions (50 μL) contained 100 ng of genomic DNA, forward and reverse primers (0.2 μM each), dNTPs (200 μM each), and Phusion High-Fidelity DNA Polymerase (1U). PCR amplification was performed as follows: 98°C^(3:00)^+[98°C^(0:15)^+50°C^(0:30)^+72°C^(0:30)^] × 25 cycles +72°C^(7:00)^. Amplified product (5 μL) from each reaction was combined and thoroughly mixed; pooled amplicons were purified by addition of Axygen AxyPrep MagPCR Clean-up beads to an equal volume of 50 μL of amplicons and incubated at room temperature for 15 minutes. Products were washed multiple times with 80% ethanol and the dried pellet resuspended in Qiagen EB Buffer (32.5 μL), incubated at room temperature for 2 minutes, and then placed on the magnetic stand for 5 minutes. The final amplicon pool was evaluated using the Advanced Analytical Fragment Analyzer automated electrophoresis system, quantified with the Qubit flourometer using the quant-iT HS dsDNA reagent kit (Invitrogen), and diluted according to Illumina’s standard protocol for sequencing on the MiSeq.

### Informatics analysis

Assembly, binning, and annotation of DNA sequences was performed at the MU Informatics Research Core Facility. Briefly, contiguous sequences of DNA were assembled using FLASH software [19], and contigs were culled if found to be short after trimming for a base quality less than 31. Qiime v1.7 [20] software was used to perform *de novo* and reference-based chimera detection and removal, and remaining contigs were assigned to operational taxonomic units (OTUs) using a criterion of 97% nucleotide identity. Taxonomy was assigned to selected OTUs using BLAST [21] against the Greengenes database [22] of 16S rRNA sequences and taxonomy. Sequence data has been uploaded to the NCBI Short Read Archive (SRA) database under accession number PRJNA263829.

### Statistical methods

All statistical analyses were performed using the R software platform with specialty Bioconductor packages as given, unless otherwise noted. Data from each time point was normalized and analyzed separately. Alpha diversity was computed based on both the Chao and Shannon methods of the raw, full data set (without pruning OTUs), and was visualized via the phyloseq package [23]. Read count tables produced by Qiime were normalized using a scaling-factor approach (cumNorm) with a data-driven target quantile as implemented in the metagenomeSeq package [24]. cumNorm is intended to correct for varying sequence depth across samples, and essentially ensures all samples have the same total number of reads. Hierarchical clustering and principal components analysis (PCA) were carried out on the log_2_-transformed normalized read counts, with one read added to all counts to avoid an undefined log. Hierarchical clustering was performed using Euclidean distance with agglomeration based on complete linkage. Rarefaction analysis was based on raw counts without normalization, which would otherwise mask the very sequence-depth effects that rarefaction plots are intended to reveal. For clarity, we emphasize that the samples were not “rarified” (i.e., random subsampling) in the sense detailed in McMurdie and Holmes [25].

Testing for interactions and main effects was implemented using a mixture-model approach that estimates the probability that an observed zero count (for a given OTU and sample) is a technical zero (caused by lack of depth) or not. This models a zero-inflated Gaussian mixture distribution and has been shown to perform best in an independent comparison against other techniques in settings similar to ours (and was the only method capable of dealing with factorial designs). Prior to statistical testing at each time point, independent filtering of OTUs was performed to improve power [26]. For each OTU, the statistical model was a 2×3 full factorial model (vendor × strain) fit via metagenomeSeq, followed by an empirical Bayes method to shrink the parameter estimates using limma [27, 28]. The testing was carried out in a hierarchical manner whereby interactions were first tested. If significant interactions were detected, pre-specified contrasts (i.e., “slicing”) were tested; otherwise, tests of each main effect were conducted and if those were significant, all pairwise comparisons were made within each main effect. The pre-specified (sliced) contrasts were all pairwise-comparisons within one factor at a fixed level of the other factor which results in a total of 9 comparisons. Phylum-level analyses was carried out in a similar manner, except that OTUs were first collapsed to the phylum level using phyloseq [23]. Finally, at each testing stage described, all p-values were adjusted for multiple testing according to method of Benjamini and Hochberg [29].

## Results

### Richness and diversity of the gut microbiota of inbred mice from different vendors

**Fig 1:**
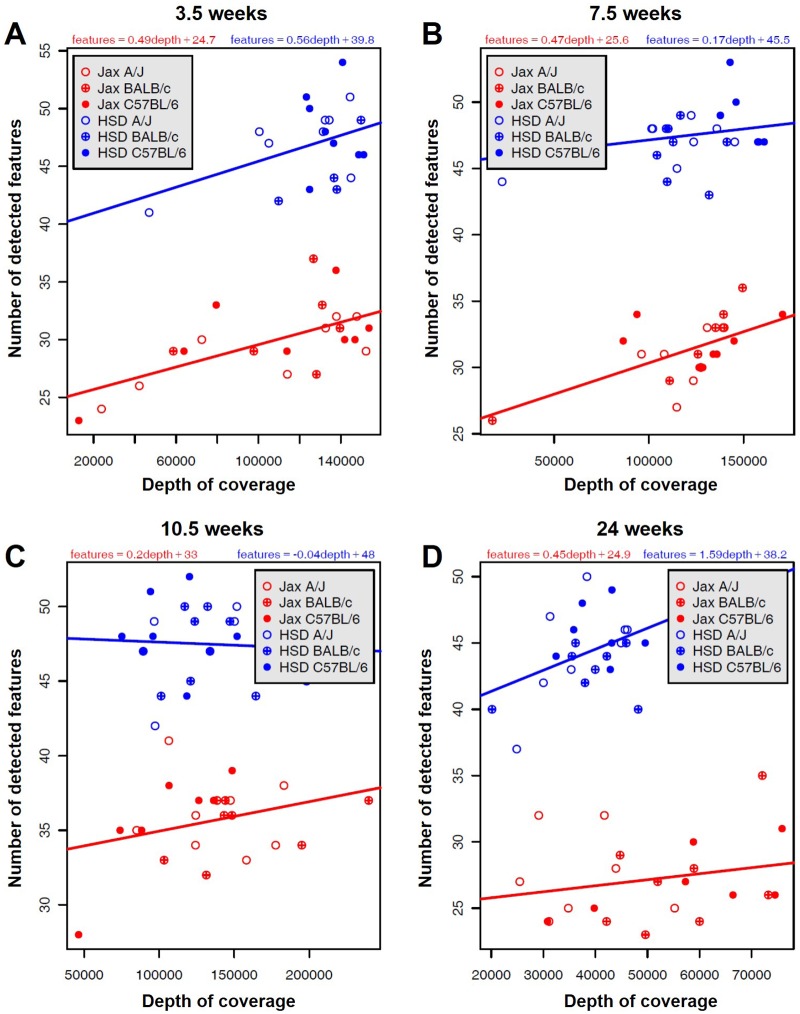
Rarefaction of sequencing data. Rarefaction analysis comparing the number of detected features to the total number of sequences obtained for each individual sample at 3.5 (A), 7.5 (B), 10.5 (C), and 24 weeks (D) of age. Data points are colored to indicate vendor and strain: *Harlan*, blue; *Jackson*, red; *A/J*, open; *BALB/c*, cross-hatched; *C57BL/6*, solid. Linear equations for mice from each vendor are shown, with depth in units of 10K reads.

**Fig 2:**
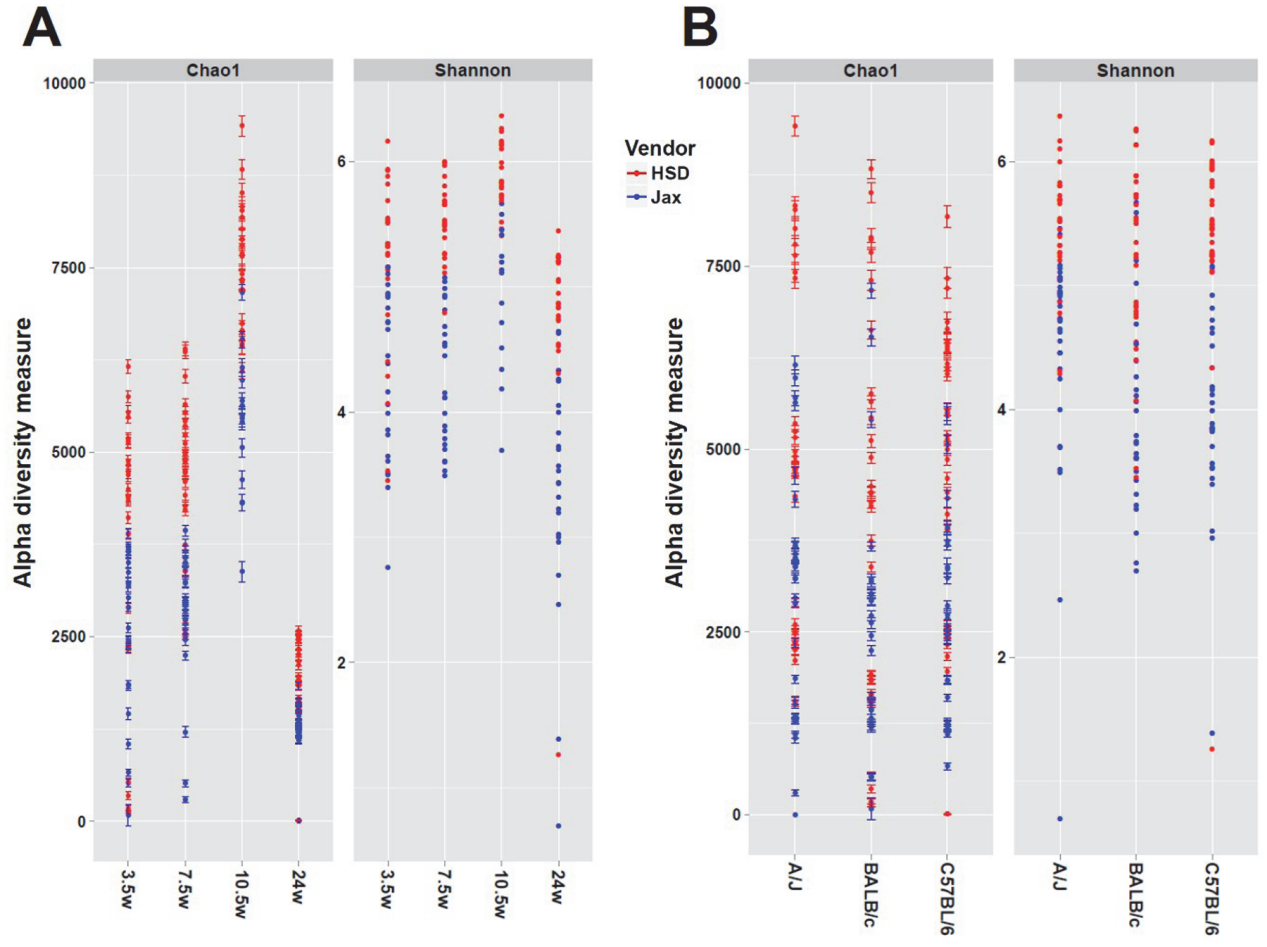
Diversity of fecal microbiota. Chao1 and Shannon estimates of microbial diversity plotted by time point (A) or by mouse strain (B). Bars on Chao1 estimates represent 95% confidence intervals. Data points are colored to indicate vendor *Harlan* (HSD), blue; *Jackson* (Jax), red.

Sequencing at a mean depth of 126462 sequences per sample, between 7909 and 19705 total unique sequences were detected among all forty-eight samples from each time point (Table 1). Assembly and binning of the raw sequence data resulted in 83 to 105 distinct operational taxonomic units (OTUs) represented at each time point. Of those, there were 58 total OTUs intersecting each time point in at least one sample. Independent (non-specific) filtering was performed to remove OTUs detected in less than eight samples per time point; filtering for sparsity resulted in analysis of between 51 and 54 OTUs at each time point. To assess adequacy of sequencing depth, rarefaction analysis was performed. The number of features detected was independent of sequencing depth above approximately 10,000 sequences per sample indicating that our sequencing depth was adequate to detect the majority of rare taxa (Fig. 1). Samples receiving less than 10,000 reads were omitted from further analyses. Unexpectedly, the rarefaction analysis demonstrated an apparent difference in the overall microbial richness in the FM of mice from the two vendors, with mice from Harlan Laboratories (HSD) clustering above mice from the Jackson Laboratory (Jax) at every time point analyzed. Comparison of the number of OTUs detected per mouse in each group confirmed that there was a significant difference in richness between vendors at each time point (*p* ≤ 0.001, 2-way ANOVA) with no detectable difference between strains. There was no significant difference in richness between different orders purchased from the same vendor. Chao1 and Shannon indices both indicated a significantly greater diversity in the FM of mice from HSD, relative to Jax (Fig. 2), and a trend toward decreased richness in the FM of 24 week old mice, relative to earlier time points. Surprisingly, there was no strain-dependent effect on gut microbial diversity.

**Table 1:** Summary statistics of sequencing data.

	3.5 weeks	7.5 weeks	10.5 weeks	24 weeks
Total # samples at > 10K reads	N = 42	N = 46	N = 48	N = 45
Mean sequencing depth (reads/sample)	117025	121560	130907	135641
Total unique sequences in all samples	11396	11815	7909	19705
Total operational taxonomic units (OTUs)	83	87	85	105
Mean # OTUs/sample ± SD				
Harlan Laboratories	51.8 ± 4.1	55.2 ± 3.6	54.8 ± 4.3	57.0 ± 4.4
The Jackson Laboratory	32.9 ± 5.0	35.6 ± 3.4	40.4 ± 3.1	41.1 ± 3.8
Common OTUs after filtering	54	54	53	51
vendor × strain interactions*	18.5% (10/54)	20.4% (11/54)	39.6% (21/53)	33.3% (17/51)
no interactions	81.5% (44/54)	79.6% (43/54)	60.4% (32/53)	66.7% (34/51)
Main effects				
vendor (adj. *p* value ≤ 0.05)	52.3% (23/44)	58.1% (25/43)	25.0% (8/32)	55.9% (19/34)
strain (adj. *p* value ≤ 0.05)	22.7% (10/44)	27.9% (12/43)	37.5% (12/32)	29.4% (10/34)

Summary of 16S rDNA amplicons sequenced using the Illumina MiSeq platform and a set of 96 bar-coded primers, and the results of testing for strain- and vendor-dependent interactions and main effects of strain and vendor on the relative abundance of consistently detected operational taxonomic units (OTUs) in the feces of 3.5, 7.5, 10.5, and 24 week old A/J, BALB/c, and C57BL/6 mice purchased from Harlan Laboratories and The Jackson Laboratory.

*Significant interactions defined as an adjusted *p* value ≤ 0.05.

### Overall similarity of the gut microbiota of inbred mice from different vendors

**Fig 3:**
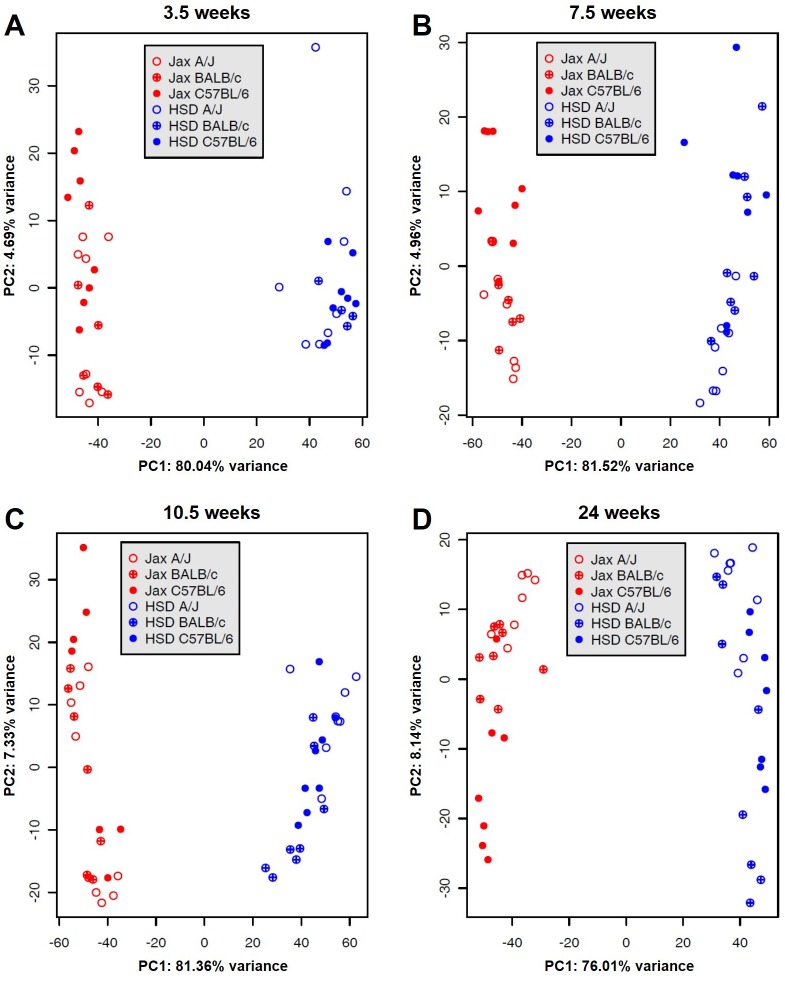
Principal component analysis. Principal component analysis of the gut microbiota in 3.5 (A), 7.5 (B), 10.5 (C), and 24 (D) week-old A/J, BALB/c, and C57BL/6 mice purchased from Harlan Laboratories or The Jackson Laboratory. Data points are colored to indicate vendor and strain: *Harlan*, blue; *Jackson*, red; *A/J*, open; *BALB/c*, cross-hatched; *C57BL/6*, solid.

To evaluate the overall similarity between the FM of mice of different genetic backgrounds or from different vendors, we performed both PCA and an agglomerative hierarchical cluster analysis. PCA is a form of dimension reduction developed to incorporate the variation and relative abundance of all detected features in a data set. Agglomerative hierarchical clustering is a multivariate statistical method of identifying natural groupings of samples by forming “most-like” pairings between individual samples, followed by subsequent joining of samples until all samples are linked. Prior to both methods, sequence abundance was normalized as previously described. PCA plots revealed two distinct vendor-specific GM compositions with no overlap between the two groups along principal component one (Fig. 3). These clusters were evident in weanling (3.5w) mice and remained throughout the duration of the study. Within each vendor cluster, the FM formed strain-dependent clusters with some overlap between strains. The strain-dependent variation was greatest along principal component two, and the associated variance increased steadily from 4.63% at 3.5 weeks to 7.94% at 24 weeks. These data suggest that the gut microbiota of laboratory mice assumes a strain-dependent structure within a larger vendor-specific range of possible compositions and that genetic background may slowly influence the composition of the FM over time, although the latter is not shown definitively.

**Fig 4:**
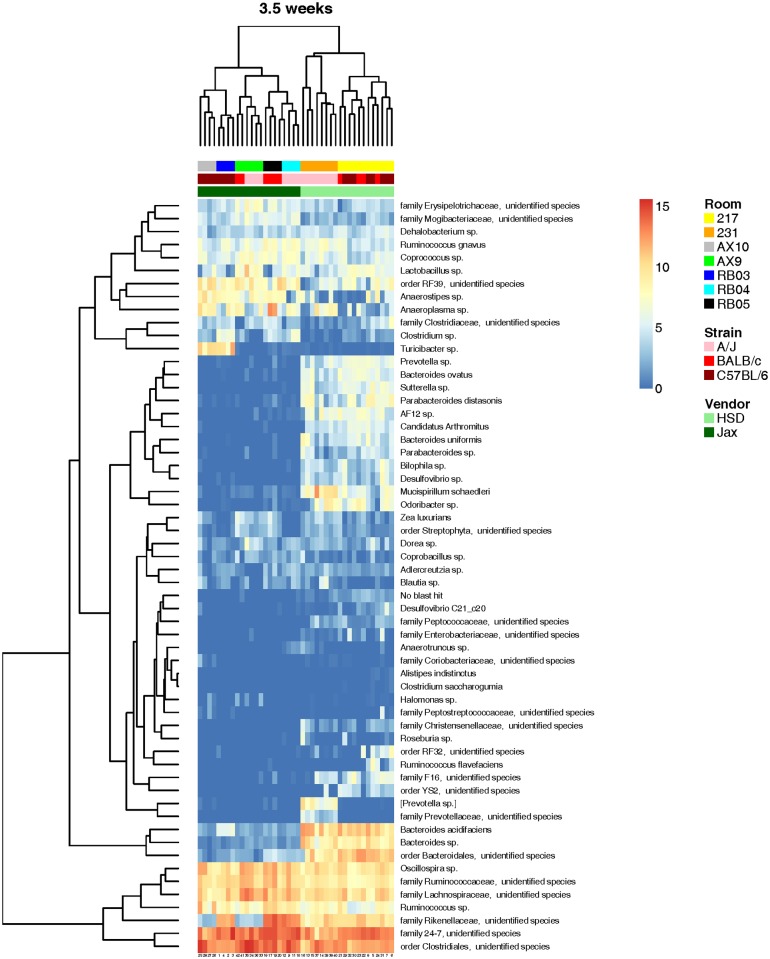
Hierarchical cluster analysis of weanling mice. Hierarchical cluster analysis of the 58 consistently detected operational taxonomic units (OTUs) in the gut microbiota of 3.5 week-old A/J, BALB/c, and C57BL/6 mice from Harlan Laboratories (HSD) and The Jackson Laboratory (Jax). Color intensity shows log_2_-transformed normalized abundance of OTUs in each sample. Color-coded bars at top indicate in ascending order: vendor, strain, and breeding room in vendor facility (legend at right). Zoom feature may be used to better visualize identity of OTUs.

**Fig 5:**
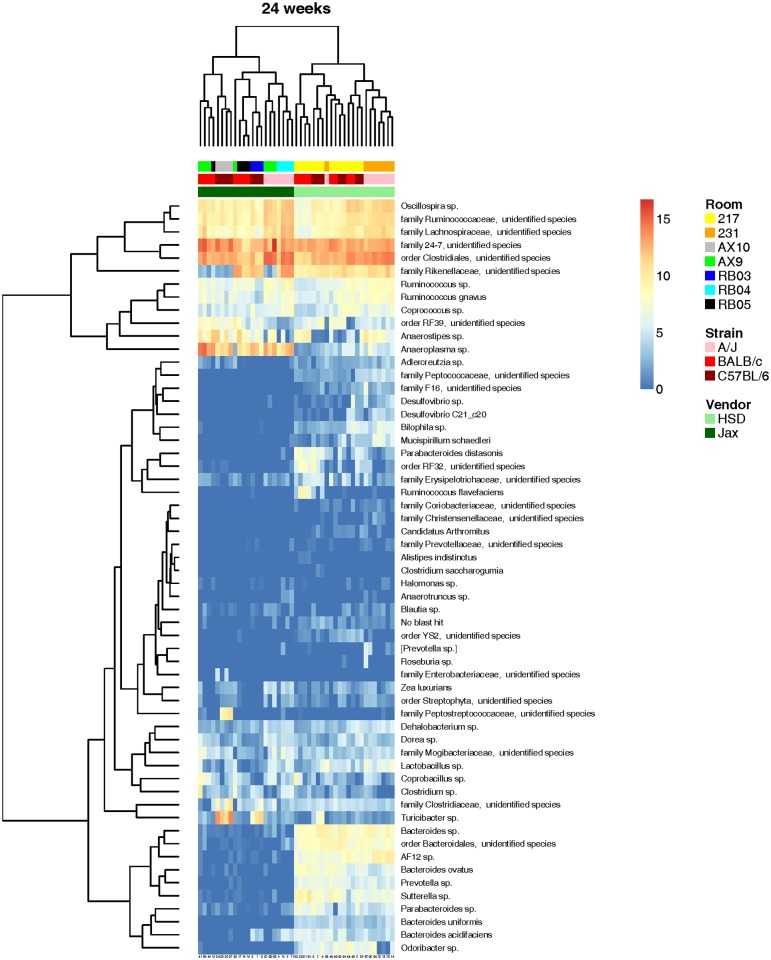
Hierarchical cluster analysis of adult mice. Hierarchical cluster analysis of the 58 consistently detected operational taxonomic units (OTUs) in the gut microbiota of 24 week-old A/J, BALB/c, and C57BL/6 mice from Harlan Laboratories (HSD) and The Jackson Laboratory (Jax). Color intensity shows log_2_-transformed normalized abundance of OTUs in each sample. Color-coded bars at top indicate in ascending order: vendor, strain, and breeding room in vendor facility (legend at right). Zoom feature may be used to better visualize identity of OTUs.

Hierarchical cluster analysis produced similar results with the primary division between vendors evident at 3.5 weeks (Fig. 4). As in the PCA, there was complete division between samples at the vendor level and, within the two vendor-dependent groups, there was clustering of mice by genetic background with some overlap between strains. We also purchased mice for these studies in two separate strain-matched orders from each vendor one week apart to assess intra-vendor variation in the FM of mice. When the production room at each vendor facility from which the mice originated was considered, there was a clustering by room of origin, even in 24w old mice (Fig. 5), suggesting that the composition of the FM of laboratory mice is determined early in life and, under routine husbandry conditions, this composition remains qualitatively unchanged until at least 24 weeks of age.

### Composition of the gut microbiota at various taxonomic levels

The FM of the mice in the current study comprised a total of nine phyla, *Actinobacteria*, *Bacteroidetes*, *Cyanobacteria*, *Deferribacteres*, *Firmicutes*, *Proteobacteria*, TM7, *Tenericutes*, and *Verrucomicrobia*. As in other mammalian hosts, *Firmicutes* and *Bacteroidetes* were the predominant phyla accounting for a combined mean abundance of between 90.8% and 96.2% at any time point. In samples collected from 3.5 week old mice, there were relatively few significant main effects of either vendor or strain on the abundance of those phyla with no detected interactions between variables, the exception being greater levels of the phylum TM7 in mice from Harlan Laboratories. Of those phyla in which significant interactions were detected, vendor-dependent differences in *Deferribacteres* and *Verrucomicrobia* were found in A/J mice, while *Proteobacteria* was more abundant in both A/J and BALB/c mice purchased from Harlan (S1 Table). Of those three phyla, strain-dependent differences were detected only in mice purchased from the Jackson Laboratory. There were several significant vendor- and strain-dependent main effects and interactions detected in samples from 7.5 week (S2 and S6 Tables) and 10.5 week (S3 and S7 Tables) old mice, however few were consistent across time points suggesting that the differences seen were at intermediate stages in the maturation of the gut microbiota. Interestingly, there were no main effects or interactions between strain and vendor detected in the abundance of any phyla at 24 weeks of age. Thus, at the phylum level, the FM matured to a relatively uniform composition in all mice examined, regardless of genetic background or source.

**Fig 6:**
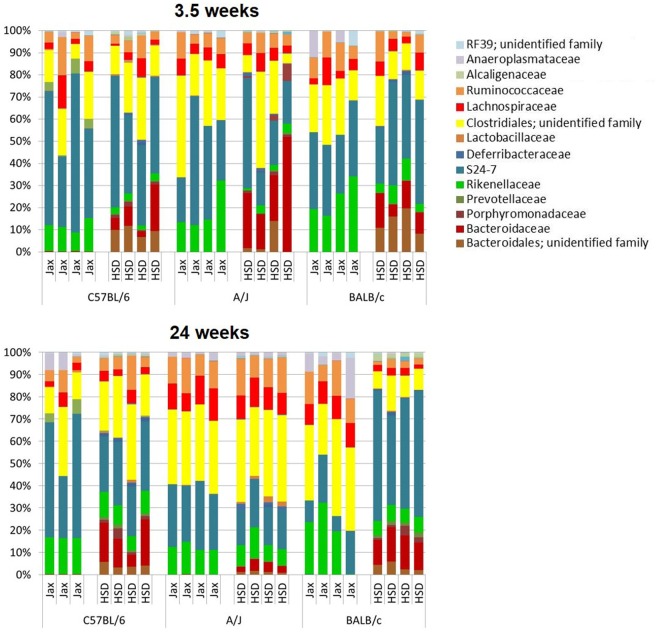
Relative abundance at taxonomic level of family. Bar charts showing the bacterial composition of the same 3.5 and 24 week old C57BL/6, A/J, and BALB/c mice purchased from Harlan Laboratories (HSD) and The Jackson Laboratory (Jax), annotated to the taxonomic level of family. Legend of prominent families is shown at right.

Annotation of sequence data to the level of class and order revealed minimal increase in taxonomic diversity, with phylum *Firmicutes* being dominated by class *Clostridia*, order *Clostridiales* and phylum *Bacteroidetes* being dominated by class *Bacteroidia*, order *Bacteroidales*. At the family level, the vendor-dependent composition of the FM became readily apparent, particularly within the order *Bacteroidales* (Fig. 6). In all strains of mice purchased from Jax, the *Bacteroidetes* were composed primarily of the families S24–7 and *Rikenellaceae*, whereas the FM of HSD mice harbored much lower levels of *Rikenellaceae* and considerably higher levels of *Bacteroidaceae*, *Porphyromonadaceae*, and other undefined family or families in the order *Bacteroidales*.

### Statistical testing for vendor- and strain-dependent differences in the abundance of consistently detected OTUs

OTUs were selected for statistical analysis based on the intersection of all four time points and independent filtering for sparsity, resulting in 54, 54, 53, and 51 OTUs at each successive time point. Taxa were first tested for interactions between vendor and strain based on an empirical Bayes F test. Those OTUs with significant interactions were then subjected to all pre-specified comparisons (“slice” contrasts) as previously described (S2 to S4 Tables).

Almost all OTUs found to be consistently different between vendors at multiple time points were more abundant in the FM of mice from Harlan Laboratories, reflecting the greater species richness in those mice (S5 to S8 Tables). Microbes in the phylum *Bacteroidetes* consistently detected in greater proportion in HSD mice included multiple *Bacteroides* spp., *Odoribacter* sp., *Parabacteroides distasonis* and *Parabacteroides* sp., *Prevotella* sp., AF12 sp., and an unresolved group of microbes in the order *Bacteroidales*.

Similarly, there were multiple OTUs from the phyla *Firmicutes* and *Proteobacteria* detected at differential abundance between vendors. As expected, segmented filamentous bacteria (SFB, Candidatus *Arthromitus*) were detected in greater abundance in the FM of HSD mice. Other OTUs in the phylum *Firmicutes* consistently found at greater levels in the FM of HSD mice include the families *Christensenellaceae* and *Peptococcaceae*, both from the order *Clostridiales*. Proteobacterial species detected in greater abundance included *Sutterella* sp., *Bilophila* sp., and *Desulfovibrio* sp.

*Mucispirillum schaedleri*, a member of the phylum *Deferribacteres*, was consistently detected at greater levels in the feces of Harlan mice although interactions between strain and vendor were present at 3.5 and 10.5 weeks of age.

While strain-dependent differences in OTU abundance were less consistent and often obscured by interactions with variable-dependent differences, there were certain OTUs that were selectively favored or limited by host genetic background. Sequences annotated to the genus *Prevotella* in the family *Paraprevotellaceae* (due to presumed polyphyly), and the family *Prevotellaceae*, were more abundant in A/JOlaHsd mice as compared to A/J mice at the three earliest collections. Interestingly, the family *Prevotellaceae* was present at significantly greater levels in A/J mice from Jackson at 24 weeks, with no strain-dependent differences detected in Harlan mice at that time point.

There were consistent strain-dependent effects on the abundance of sequences resolved to the family *Clostridiaceae* with C57BL/6 mice harboring purportedly more microbes than A/J mice at 3.5, 7.5, and 10.5 weeks, and BALB/c showing an intermediate degree of colonization. At 24 weeks, interactions detected between variables indicated that C57BL/6, but not A/J or BALB/c, mice harbored significant vendor-dependent differences in the number of *Clostridiaceae*-specific sequences. Similarly, no strain-dependent differences were detected in pairwise comparisons within Harlan mice while, within Jackson mice, C57BL/6 mice harbored significantly greater levels of family *Clostridiaceae* than A/J or BALB/c mice, collectively suggesting that C57BL/6J mice are preferentially colonized by this taxon.

## Discussion

With the advent of culture-independent techniques, it is now possible to characterize the FM of laboratory animals in great detail. Such methodologies will allow for trouble-shooting of altered model phenotypes and, potentially, refinement of affected models. The reproducibility of biomedical research, particularly pre-clinical research using animal models, has recently become a focus point for the National Institutes of Health (NIH) [4, 5]. Potential reasons often cited for poor reproducibility include lack of randomization, effects of sex differences, coincidental findings related to poor experimental power, and vague environmental differences between laboratories. We propose that variation in the FM of experimental animals is one of those environmental differences contributing to poor reproducibility of studies using animal models.

Differences in the composition of the FM have been implicated in a broad spectrum of human diseases and conditions and similar mechanisms likely affect animal models of those diseases. Recently, several groups have demonstrated FM-dependent model phenotypes due to the heterogeneity of the FM in mice from different commercial vendors [6–8, 10, 30]. In these studies, differences in disease severity were attributed to the presence or absence of segmented filamentous bacteria, long-recognized commensal gut microbes residing in the ileum of mice [31]. Notably, mice from the Jackson Laboratory are free of SFB while most mice from other large commercial vendors are colonized with SFB. While SFB causes no detectable inflammation in wild-type mice, the studies above highlight the ability of individual commensal species to influence the development or function of the host immune system such that, in the context of a disease model, disease severity is modulated.

Similarly, other groups have demonstrated FM-dependent differences in disease severity not attributable to SFB [9, 32–34], implicating the FM as a nearly ubiquitous variable in biomedical research using mouse models. We detected significant vendor-dependent differences in the abundance of several OTUs of potential consequence to phenotypic differences and poor reproducibility of studies when using mice from different sources, or even mice exposed to the FM of mice from other vendors.

Mice from HSD harbored significantly greater levels of several OTUs in the phylum *Bacteroidetes*, including multiple *Bacteroides* spp. and *Parabacteroides* spp. Antigens derived from multiple microbes in these genera, including *Parabacteroides distasonis*, have been shown to induce the differentiation of colonic FoxP3^+^ T_reg_ cells [35, 36], and administration of *P*. *distasonis*-derived antigens ameliorates the severity of DSS-induced colitis [37]. Interestingly, *P*. *distasonis*, detected at significantly greater levels in mice from HSD, is a component of the altered Schaedler flora, used by most vendors to inoculate germ-free founder animals when establishing a new production colony. While it is thus not surprising to detect this organism in all mice tested, its differential abundance speaks to the ability of the surrounding microbial milieu and other environmental factors to shape the FM.

Closely related Gram negative microbes in the class *Deltaproteobacteria*, *Bilophila* sp. and *Desulfovibrio* spp., were also detected at significantly greater levels in mice from HSD. *Bilophila* sp., a common inhabitant of the human GI tract, can adhere to human cells [38] and displays endotoxin activity [39]. It is a common isolate in cases of perforated appendicitis, and has been associated with a wide range of clinical infections in multiple tissues. *Desulfovibrio* spp. are sulfate-reducing microbes rarely isolated from clinical cases, also expressing a variety of lipopolysaccharide molecules. Additionally, *Sutterella* sp. (class *Betaproteobacteria*) was detected at significantly greater levels in the FM of mice from Harlan. This species has recently gained attention for its possible link to autism and common gastrointestinal symptoms associated with autism [40].

Previously, multiple groups have used molecular finger-printing techniques to demonstrate institutional or vendor-specific gut microbial communities [13, 14]. However, these methods provide no information regarding the identity of microbes detected or overall species richness or diversity, all factors in susceptibility to certain human enteric diseases [41]. Studies to date employing next-generation approaches to characterize the FM of laboratory mice [42, 43] have not examined vendor-dependent differences. Moreover, it must be recognized that many researchers breed experimental mice “in house”, begging the question of inter-institutional differences in the FM. Herein, we demonstrate strain- and vendor-dependent differences detected within one institution and it must be acknowledged that the same study performed under different husbandry conditions or in a different geographical location would potentially yield different results, particularly in samples collected from mice that have been housed for extended periods.

Factors affecting the composition of the murine FM include multiple genetic regions, sex, diet, age, and a host of other subtle and likely unrecognized environmental stimuli. In general, host genetic background is relatively strong determinant of FM composition [11, 12]. In the present study, analysis of mice of multiple genetic backgrounds from more than one vendor allowed the identification of strain-dependent selective colonization by specific microbes or higher taxa. Further studies are needed to characterize the relationship between the genetic factors conferring selection for certain taxa and the potential to use those associations diagnostically or therapeutically. Additionally, the current study was performed solely in female mice, to allow for long-term co-housing to reduce costs. While we have seen negligible differences between the FM of littermate male and female mice (unpublished), additional studies are needed to validate this observation statistically.

The differences detected between vendors in microbial richness and diversity may have use as a facet of modeling diseases such as inflammatory bowel disease and colorectal cancer, in the context of a complex FM. Mice of the desired genetic background, such as IL-10^-/-^ or Smad3^-/-^ mice, could be rederived via embryo transfer to surrogate dams harboring a Jackson or Harlan FM. The fact that mouse pups derive their FM primarily from the dam raises another consideration. When rederiving mice into new research facilities, as is common practice to avoid the introduction of overt and opportunistic pathogens, the source of surrogate dams may impact model performance. These and similar issues underlie our expanding appreciation of the impact of the FM on host health.

At the most basic level, these data highlight the need to acknowledge vendor-dependent differences in the composition of the FM of mice. It is our hope that, as the accessibility of NGS technology increases, additional studies will further our knowledge of the FM of laboratory animals. Unlike the standardization of genetics achieved via the creation of inbred mice, it would be very difficult, if not impossible, to standardize the FM of mice used in biomedical research. Rather, it is ideal to characterize it to the best on one’s abilities, recognize that it may play a role in a model phenotype, and be cognizant of factors which may influence its composition.

## Supporting Information

S1 Table Pairwise comparisons of phyla with interactions. Pairwise comparisons within variables of the relative abundance of phyla with detected interactions between strain and vendor at each time point. Log fold difference between groups (logFC), calculated *p* values (P.Value), and *p* values adjusted to control false discovery (adj.P.Val) are shown. Adjusted *p* values below 0.05 are shaded in grey.
(PDF)

S2 Table Pairwise comparisons of operational taxonomic units with interactions at 3.5 weeks. Pairwise comparisons within variables of the relative abundance of operational taxonomic units (OTUs) with detected interactions between strain and vendor at 3.5 weeks of age. Log-fold difference between groups (logFC), calculated *p* values (P.Value), and *p* values adjusted to control false discovery (adj.P.Val) are shown. Adjusted *p* values below 0.05 are shaded in grey. Genus names in square brackets are annotations supplied by the Greengenes database and not officially accepted by the Society for General Microbiology, typically due to polyphyly of the genus.
(PDF)

S3 Table Pairwise comparisons of operational taxonomic units with interactions at 7.5 weeks. Pairwise comparisons within variables of the relative abundance of operational taxonomic units (OTUs) with detected interactions between strain and vendor at 7.5 weeks of age. Log-fold difference between groups (logFC), calculated *p* values (P.Value), and *p* values adjusted to control false discovery (adj.P.Val) are shown. Adjusted *p* values below 0.05 are shaded in grey. Genus names in square brackets are annotations supplied by the Greengenes database and not officially accepted by the Society for General Microbiology, typically due to polyphyly of the genus.
(PDF)

S4 Table Pairwise comparisons of operational taxonomic units with interactions at 10.5 weeks. Pairwise comparisons within variables of the relative abundance of operational taxonomic units (OTUs) with detected interactions between strain and vendor at 10.5 weeks of age. Log-fold difference between groups (logFC), calculated *p* values (P.Value), and *p* values adjusted to control false discovery (adj.P.Val) are shown. Adjusted *p* values below 0.05 are shaded in grey. Genus names in square brackets are annotations supplied by the Greengenes database and not officially accepted by the Society for General Microbiology, typically due to polyphyly of the genus.
(PDF)

S5 Table Main effects of vendor and genetic background at 3.5 weeks. Testing for vendor- and strain-dependent main effects on the relative abundance of phyla and operational taxonomic units (OTUs) with no interactions between variables in 3.5 week old A/J, BALB/c, and C57BL/6 mice purchased from Harlan Laboratories (HSD) and The Jackson Laboratory (Jax). Log-normalized average abundance (AveExpr) of each OTU (averaged across all samples), log_2_ fold difference between groups (logFC), calculated *p* values (P.Value), and adjusted *p* values (adj.P.Val) are shown. Adjusted *p* values below 0.05 are shaded in grey. Taxon names above the rank of genus in square brackets are names proposed by the Greengenes curators and will not be found in NCBI. Genus names in square brackets are annotations supplied by the Greengenes database and not officially accepted by the Society for General Microbiology, typically due to polyphyly of the genus.
(PDF)

S6 Table Main effects of vendor and genetic background at 7.5 weeks. Testing for vendor- and strain-dependent main effects on the relative abundance of phyla and operational taxonomic units (OTUs) with no interactions between variables in 7.5 week old A/J, BALB/c, and C57BL/6 mice purchased from Harlan Laboratories (HSD) and The Jackson Laboratory (Jax). Log-normalized average abundance (AveExpr) of each OTU (averaged across all samples), log_2_ fold difference between groups (logFC), calculated *p* values (P.Value), and adjusted *p* values (adj.P.Val) are shown. Adjusted *p* values below 0.05 are shaded in grey. Taxon names above the rank of genus in square brackets are names proposed by the Greengenes curators and will not be found in NCBI. Genus names in square brackets are annotations supplied by the Greengenes database and not officially accepted by the Society for General Microbiology, typically due to polyphyly of the genus.
(PDF)

S7 Table Main effects of vendor and genetic background at 10.5 weeks. Testing for vendor- and strain-dependent main effects on the relative abundance of phyla and operational taxonomic units (OTUs) with no interactions between variables in 10.5 week old A/J, BALB/c, and C57BL/6 mice purchased from Harlan Laboratories (HSD) and The Jackson Laboratory (Jax). Log-normalized average abundance (AveExpr) of each OTU (averaged across all samples), log_2_ fold difference between groups (logFC), calculated *p* values (P.Value), and adjusted *p* values (adj.P.Val) are shown. Adjusted *p* values below 0.05 are shaded in grey. Taxon names above the rank of genus in square brackets are names proposed by the Greengenes curators and will not be found in NCBI. Genus names in square brackets are annotations supplied by the Greengenes database and not officially accepted by the Society for General Microbiology, typically due to polyphyly of the genus.
(PDF)

S8 Table Main effects of vendor and genetic background at 24 weeks. Testing for vendor- and strain-dependent main effects on the relative abundance of phyla and operational taxonomic units (OTUs) with no interactions between variables in 24 week old A/J, BALB/c, and C57BL/6 mice purchased from Harlan Laboratories (HSD) and The Jackson Laboratory (Jax). Log-normalized average abundance (AveExpr) of each OTU (averaged across all samples), log_2_ fold difference between groups (logFC), calculated *p* values (P.Value), and adjusted *p* values (adj.P.Val) are shown. Adjusted *p* values below 0.05 are shaded in grey. Taxon names above the rank of genus in square brackets are names proposed by the Greengenes curators and will not be found in NCBI. Genus names in square brackets are annotations supplied by the Greengenes database and not officially accepted by the Society for General Microbiology, typically due to polyphyly of the genus.
(PDF)
