# Supplementary material for: Effects of Vendor and Genetic Background on the Composition of the Fecal Microbiota of Inbred Mice
Source: PLoS One. 2015 Feb 12;10(2):e0116704. doi: 10.1371/journal.pone.0116704 (PMC4326421; doi:10.1371/journal.pone.0116704)
Supplement: S1 Table — Pairwise comparisons within variables of the relative abundance of phyla with detected interactions between strain and vendor at each time point. Log fold difference between groups (logFC), calculated p values (P.Value), and p values adjusted to control false discovery (adj.P.Val) are shown. Adjusted p values below 0.05 are shaded in grey. (PDF) [file pone.0116704.s001.pdf]

Phyla with interactions at 3.5 weeks	Within A/J			Within BALB/c			Within C57BL/6		
	HSD relative to Jax			HSD relative to Jax			HSD relative to Jax		
**Phylum**	**logFC**	**P.Value**	**adj.P.Val**	**logFC**	**P.Value**	**adj.P.Val**	**logFC**	**P.Value**	**adj.P.Val**
Deferribacteres	2.611791	0.00238	0.00238	-1.11316	0.29728	0.44592	-0.50667	0.560694	0.835016
Proteobacteria	3.586889	5.48E-07	1.64E-06	4.30041	1.11E-06	3.33E-06	1.16702	0.051855	0.155565
Verrucomicrobia	-2.99403	0.000142	0.000213	-0.01302	0.986226	0.986226	0.122861	0.835016	0.835016
	**Within HSD**								
	**A/J relative to BALB/c**			**C57BL/6 relative to A/J**			**C57BL/6 relative to BALB/c**		
**Phylum**	**logFC**	**P.Value**	**adj.P.Val**	**logFC**	**P.Value**	**adj.P.Val**	**logFC**	**P.Value**	**adj.P.Val**
Deferribacteres	2.109992	0.045021	0.135064	-1.14806	0.190241	0.216834	0.961928	0.368812	0.890817
Proteobacteria	-0.6366	0.377639	0.377639	0.735225	0.216834	0.216834	0.098621	0.890817	0.890817
Verrucomicrobia	-1.27982	0.107879	0.161818	1.583652	0.032274	0.096822	0.303836	0.667281	0.890817
	**Within Jax**								
	**A/J relative to BALB/c**			**C57BL/6 relative to A/J**			**C57BL/6 relative to BALB/c**		
**Phylum**	**logFC**	**P.Value**	**adj.P.Val**	**logFC**	**P.Value**	**adj.P.Val**	**logFC**	**P.Value**	**adj.P.Val**
Deferribacteres	-1.61496	0.060751	0.091127	1.970396	0.017926	0.017926	0.355433	0.683335	0.795138
Proteobacteria	0.076917	0.904401	0.904401	3.155093	3.33E-06	9.98E-06	3.232011	7.31E-06	2.19E-05
Verrucomicrobia	1.701194	0.016537	0.049611	-1.53324	0.012735	0.017926	0.167957	0.795138	0.795138

Phyla with interactions at 7.5 weeks	Within A/J			Within BALB/c			Within C57BL/6		
	HSD relative to Jax			HSD relative to Jax			HSD relative to Jax		
**Phylum**	**logFC**	**P.Value**	**adj.P.Val**	**logFC**	**P.Value**	**adj.P.Val**	**logFC**	**P.Value**	**adj.P.Val**
Bacteroidetes	-0.65153	0.046416	0.185664	-0.42808	0.288243	0.288243	-1.78642	0.000377	0.000753
Proteobacteria	-0.74012	0.275543	0.435756	-1.28991	0.131653	0.175537	-4.33494	6.11E-05	0.000245
Tenericutes	-0.48148	0.326817	0.435756	1.179563	0.058896	0.117793	1.671782	0.022759	0.030345
Verrucomicrobia	0.282622	0.67599	0.67599	-3.13391	0.000371	0.001482	0.51888	0.583066	0.583066
	**Within HSD**								
	**A/J relative to BALB/c**			**C57BL/6 relative to A/J**			**C57BL/6 relative to BALB/c**		
**Phylum**	**logFC**	**P.Value**	**adj.P.Val**	**logFC**	**P.Value**	**adj.P.Val**	**logFC**	**P.Value**	**adj.P.Val**
Bacteroidetes	0.175955	0.517141	0.517141	-0.87547	0.009142	0.018284	-0.69952	0.02659	0.035453
Proteobacteria	0.942912	0.104109	0.416437	-3.07649	4.27E-05	0.000171	-2.13358	0.001821	0.007285
Tenericutes	-0.43596	0.295244	0.517141	0.958093	0.05707	0.076094	0.522136	0.268216	0.268216
Verrucomicrobia	0.378574	0.500041	0.517141	1.109539	0.089275	0.089275	1.488113	0.019123	0.035453
	**Within Jax**								
	**A/J relative to BALB/c**			**C57BL/6 relative to A/J**			**C57BL/6 relative to BALB/c**		
**Phylum**	**logFC**	**P.Value**	**adj.P.Val**	**logFC**	**P.Value**	**adj.P.Val**	**logFC**	**P.Value**	**adj.P.Val**
Bacteroidetes	0.399405	0.193834	0.258445	0.259423	0.36047	0.385971	0.658828	0.024156	0.048313
Proteobacteria	0.393122	0.54111	0.54111	0.518329	0.385971	0.385971	0.911451	0.132632	0.176843
Tenericutes	1.225088	0.011151	0.022301	-1.19517	0.007819	0.031277	0.029918	0.944961	0.944961
Verrucomicrobia	-3.03796	7.35E-06	2.94E-05	0.873281	0.142166	0.284332	-2.16468	6.1E-05	0.000244

Phyla with interactions at 10.5 weeks	Within A/J			Within BALB/c			Within C57BL/6		
	HSD relative to Jax			HSD relative to Jax			HSD relative to Jax		
**Phylum**	**logFC**	**P.Value**	**adj.P.Val**	**logFC**	**P.Value**	**adj.P.Val**	**logFC**	**P.Value**	**adj.P.Val**
Cyanobacteria	0.304715	0.713057	0.714692	2.122697	0.013111	0.019666	3.744766	1.8E-05	1.8E-05
Deferribacteres	8.363844	3.08E-16	9.24E-16	4.468571	8.3E-05	0.000249	6.159187	1.26E-09	3.78E-09
Verrucomicrobia	-0.40903	0.714692	0.714692	0.737518	0.546507	0.546507	5.566851	4.69E-06	7.04E-06
	**Within HSD**								
	**A/J relative to BALB/c**			**C57BL/6 relative to A/J**			**C57BL/6 relative to BALB/c**		
**Phylum**	**logFC**	**P.Value**	**adj.P.Val**	**logFC**	**P.Value**	**adj.P.Val**	**logFC**	**P.Value**	**adj.P.Val**
Cyanobacteria	-1.71962	0.043463	0.065194	1.176648	0.160507	0.160507	-0.54297	0.507971	0.507971
Deferribacteres	4.038477	1.62E-05	4.87E-05	-2.23053	0.013467	0.0202	1.807951	0.038946	0.058418
Verrucomicrobia	-0.7268	0.540282	0.540282	5.915911	9.29E-07	2.79E-06	5.189114	1.32E-05	3.97E-05
	**Within Jax**								
	**A/J relative to BALB/c**			**C57BL/6 relative to A/J**			**C57BL/6 relative to BALB/c**		
**Phylum**	**logFC**	**P.Value**	**adj.P.Val**	**logFC**	**P.Value**	**adj.P.Val**	**logFC**	**P.Value**	**adj.P.Val**
Cyanobacteria	0.098365	0.905498	0.905498	-2.2634	0.007804	0.023411	-2.16504	0.011389	0.034168
Deferribacteres	0.143204	0.89291	0.905498	-0.02587	0.977884	0.977884	0.117335	0.914069	0.914069
Verrucomicrobia	0.419747	0.716648	0.905498	-0.05997	0.957974	0.977884	0.35978	0.765898	0.914069

Table S1
