# Supplementary material for: Effects of Vendor and Genetic Background on the Composition of the Fecal Microbiota of Inbred Mice
Source: PLoS One. 2015 Feb 12;10(2):e0116704. doi: 10.1371/journal.pone.0116704 (PMC4326421; doi:10.1371/journal.pone.0116704)
Supplement: S2 Table — Pairwise comparisons within variables of the relative abundance of operational taxonomic units (OTUs) with detected interactions between strain and vendor at 3.5 weeks of age. Log-fold difference between groups (logFC), calculated p values (P.Value), and p values adjusted to control false discovery (adj.P.Val) are shown. Adjusted p values below 0.05 are shaded in grey. Genus names in square brackets are annotations supplied by the Greengenes database and not officially accepted by the Society for General Microbiology, typically due to polyphyly of the genus. (PDF) [file pone.0116704.s002.pdf]

OTUs with interactions at 3.5 weeks			Within A/J			Within BALB/c			Within C57BL/6		
			HSD relative to Jax			HSD relative to Jax			HSD relative to Jax		
**Phylum**	**Family**	**Operational taxonomic unit (OTU)**	**logFC**	**P.Value**	**adj.P.Val**	**logFC**	**P.Value**	**adj.P.Val**	**logFC**	**P.Value**	**adj.P.Val**
Actinobacteria	*Coriobacteriaceae*	family *Coriobacteriaceae* , unidentified species	1.544669	0.026048	0.037212	0.230062	0.746326	0.746326	-0.80167	0.189702	0.21078
Bacteroidetes	[*Paraprevotellaceae*]	[*Prevotella*] sp.	8.473077	7.60E-14	7.60E-13	-1.27334	0.148649	0.297298	-1.37612	0.062743	0.089633
Bacteroidetes	*Prevotellaceae*	family *Prevotellaceae* , unidentified species	4.799868	1.98E-08	6.59E-08	-0.88239	0.298914	0.366755	-1.00043	0.138171	0.172713
Bacteroidetes		family 24-7, unidentified species	-2.83826	9.93E-05	0.000199	-0.73526	0.305809	0.366755	-0.36718	0.533067	0.533067
Bacteroidetes		order *Bacteroidales*	6.040287	2.76E-07	6.90E-07	8.483672	8.25E-10	8.25E-09	9.542037	2.64E-13	1.32E-12
Deferribacteres	*Deferribacteraceae*	*Mucispirillum schaedleri*	10.50942	1.45E-12	7.26E-12	5.460973	1.26E-05	6.30E-05	6.996205	3.17E-09	7.92E-09
Firmicutes	*Lactobacillaceae*	*Lactobacillus* sp.	-3.35017	0.009038	0.015063	1.309017	0.33008	0.366755	3.330277	0.004109	0.008219
Firmicutes	*Turicibacteraceae*	*Turicibacter* sp.	-0.01138	0.984209	0.984209	-0.75191	0.225864	0.366755	-10.7035	5.70E-23	5.70E-22
Proteobacteria		order RF32, unidentified species	2.182788	0.03611	0.045137	5.327961	4.11E-05	0.000137	8.387148	8.77E-11	2.92E-10
Tenericutes	*Anaeroplasmataceae*	*Anaeroplasma* sp.	1.267817	0.511957	0.568841	-5.69502	0.009419	0.023549	-4.52469	0.012047	0.020078
			Within HSD								
			A/J relative to BALB/c			C57BL/6 relative to A/J			C57BL/6 relative to BALB/c		
**Phylum**	**Family**	**Operational taxonomic unit (OTU)**	**logFC**	**P.Value**	**adj.P.Val**	**logFC**	**P.Value**	**adj.P.Val**	**logFC**	**P.Value**	**adj.P.Val**
Actinobacteria	*Coriobacteriaceae*	family *Coriobacteriaceae* , unidentified species	1.25153	0.059626	0.080602	-0.18219	0.731897	0.731897	1.069343	0.111506	0.557528
Bacteroidetes	[*Paraprevotellaceae*]	[*Prevotella*] sp.	9.074513	2.32E-14	2.32E-13	-9.76388	5.37E-18	5.37E-17	-0.68936	0.38958	0.574027
Bacteroidetes	*Prevotellaceae*	family *Prevotellaceae* , unidentified species	5.922087	6.54E-10	3.27E-09	-6.48406	2.09E-13	1.04E-12	-0.56198	0.45843	0.574027
Bacteroidetes		family 24-7, unidentified species	-2.22798	0.001259	0.002517	1.535588	0.005355	0.008925	-0.69239	0.293413	0.574027
Bacteroidetes		order *Bacteroidales*	-3.67535	0.000409	0.001023	2.453469	0.003099	0.006199	-1.22188	0.213924	0.574027
Deferribacteres	*Deferribacteraceae*	*Mucispirillum schaedleri*	4.524538	5.30E-05	0.000177	-3.32946	0.000224	0.00056	1.195083	0.24571	0.574027
Firmicutes	*Lactobacillaceae*	*Lactobacillus* sp.	-2.24322	0.067683	0.080602	2.33366	0.021118	0.030169	0.090439	0.940958	0.940958
Firmicutes	*Turicibacteraceae*	*Turicibacter* sp.	0.7302	0.198588	0.198588	-0.32047	0.472557	0.525063	0.409729	0.459222	0.574027
Proteobacteria		order RF32, unidentified species	-2.84493	0.007406	0.012343	6.586808	1.72E-09	5.72E-09	3.741876	0.000965	0.009646
Tenericutes	*Anaeroplasmataceae*	*Anaeroplasma* sp.	3.467175	0.072542	0.080602	-3.29133	0.037484	0.046854	0.175843	0.927097	0.940958
			Within Jax								
			A/J relative to BALB/c			C57BL/6 relative to A/J			C57BL/6 relative to BALB/c		
**Phylum**	**Family**	**Operational taxonomic unit (OTU)**	**logFC**	**P.Value**	**adj.P.Val**	**logFC**	**P.Value**	**adj.P.Val**	**logFC**	**P.Value**	**adj.P.Val**
Actinobacteria	*Coriobacteriaceae*	family *Coriobacteriaceae* , unidentified species	-0.06308	0.910325	0.982705	2.164156	0.000211	0.000703	2.101079	0.000509	0.002547
Bacteroidetes	[*Paraprevotellaceae*]	[*Prevotella*] sp.	-0.67191	0.330579	0.826446	0.085316	0.897673	0.897673	-0.58659	0.374207	0.617833
Bacteroidetes	*Prevotellaceae*	family *Prevotellaceae* , unidentified species	0.239826	0.726261	0.982705	-0.68376	0.305207	0.436011	-0.44394	0.520034	0.617833
Bacteroidetes		family 24-7, unidentified species	-0.12498	0.825003	0.982705	-0.9355	0.096038	0.240096	-1.06047	0.07059	0.162689
Bacteroidetes		order *Bacteroidales*	-1.23197	0.150187	0.500623	-1.04828	0.229902	0.383169	-2.28025	0.013289	0.044296
Deferribacteres	*Deferribacteraceae*	*Mucispirillum schaedleri*	-0.5239	0.554661	0.982705	0.183755	0.844463	0.897673	-0.34015	0.720537	0.720537
Firmicutes	*Lactobacillaceae*	*Lactobacillus* sp.	2.415965	0.030612	0.203466	-4.34679	0.000136	0.000681	-1.93082	0.081344	0.162689
Firmicutes	*Turicibacteraceae*	*Turicibacter* sp.	-0.01033	0.982705	0.982705	10.37164	9.69E-24	9.69E-23	10.36131	1.39E-23	1.39E-22
Proteobacteria		order RF32, unidentified species	0.300241	0.770867	0.982705	0.382449	0.680584	0.85073	0.68269	0.491388	0.617833
Tenericutes	*Anaeroplasmataceae*	*Anaeroplasma* sp.	-3.49567	0.040693	0.203466	2.50117	0.12866	0.257321	-0.9945	0.556049	0.617833

Table S2
