# Supplementary material for: Effects of Vendor and Genetic Background on the Composition of the Fecal Microbiota of Inbred Mice
Source: PLoS One. 2015 Feb 12;10(2):e0116704. doi: 10.1371/journal.pone.0116704 (PMC4326421; doi:10.1371/journal.pone.0116704)
Supplement: S3 Table — Pairwise comparisons within variables of the relative abundance of operational taxonomic units (OTUs) with detected interactions between strain and vendor at 7.5 weeks of age. Log-fold difference between groups (logFC), calculated p values (P.Value), and p values adjusted to control false discovery (adj.P.Val) are shown. Adjusted p values below 0.05 are shaded in grey. Genus names in square brackets are annotations supplied by the Greengenes database and not officially accepted by the Society for General Microbiology, typically due to polyphyly of the genus. (PDF) [file pone.0116704.s003.pdf]

OTUs with interactions at 7.5 weeks			Within A/J			Within BALB/c			Within C57BL/6		
			HSD relative to Jax			HSD relative to Jax			HSD relative to Jax		
**Phylum**	**Family**	**Operational taxonomic unit (OTU)**	**logFC**	**P.Value**	**adj.P.Val**	**logFC**	**P.Value**	**adj.P.Val**	**logFC**	**P.Value**	**adj.P.Val**
Actinobacteria	*Coriobacteriaceae*	family *Coriobacteriaceae* , unidentified species	1.053479	0.087295	0.120031	0.908905	0.145202	0.228175	3.004926	3.50E-06	7.69E-06
Bacteroidetes	[*Paraprevotellaceae*]	[*Prevotella*] sp.	3.265139	0.000587	0.002298	-2.648014	0.007683	0.014086	-2.730894	0.005562	0.007647
Bacteroidetes	*Prevotellaceae*	family *Prevotellaceae* , unidentified species	2.610356	0.008293	0.015204	-1.048077	0.251986	0.307983	-1.468308	0.118773	0.145168
Cyanobacteria		order *Streptophyta* , unidentified species	-2.384911	0.00645	0.01419	1.226093	0.176441	0.242607	-0.105024	0.903867	0.903867
Firmicutes	*Christensenellaceae*	family *Christensenellaceae* , unidentified species	1.69794	0.000627	0.002298	1.692761	0.000717	0.001577	3.273838	2.45E-09	6.73E-09
Firmicutes	*Lachnospiraceae*	*Roseburia* sp.	1.544669	0.184993	0.203493	-5.143432	0.000355	0.000977	1.707865	0.189521	0.208473
Firmicutes	*Peptostreptococcaceae*	family *Peptostreptococcaceae* , unidentified species	0.327958	0.661824	0.661824	0.240685	0.776134	0.776134	-4.183582	9.65E-06	1.77E-05
Firmicutes	*Ruminococcaceae*	*Ruminococcus flavefaciens*	-1.148423	0.060876	0.095662	4.427253	4.93E-10	1.81E-09	5.763824	1.95E-15	7.14E-15
Firmicutes	*Turicibacteraceae*	*Turicibacter* sp.	0.977124	0.137957	0.168614	0.685423	0.327229	0.359952	-8.504613	2.32E-16	1.28E-15
Proteobacteria	*Alcaligenaceae*	*Sutterella* sp.	4.802763	2.85E-10	3.14E-09	7.029625	3.17E-14	3.48E-13	8.191755	2.75E-17	3.03E-16
Proteobacteria	*Enterobacteriaceae*	family *Enterobacteriaceae* , unidentified species	2.191445	0.004033	0.011091	6.905207	3.29E-12	1.81E-11	2.631698	0.000513	0.000806
			Within HSD								
			A/J relative to BALB/c			C57BL/6 relative to A/J			C57BL/6 relative to BALB/c		
**Phylum**	**Family**	**Operational taxonomic unit (OTU)**	**logFC**	**P.Value**	**adj.P.Val**	**logFC**	**P.Value**	**adj.P.Val**	**logFC**	**P.Value**	**adj.P.Val**
Actinobacteria	*Coriobacteriaceae*	family *Coriobacteriaceae* , unidentified species	0.167727	0.735805	0.809385	3.468362	6.87E-09	2.52E-08	3.636089	1.11E-09	1.23E-08
Bacteroidetes	[*Paraprevotellaceae*]	[*Prevotella*] sp.	6.264923	1.18E-10	6.47E-10	-6.957244	2.33E-11	1.28E-10	-0.692321	0.371273	0.5105
Bacteroidetes	*Prevotellaceae*	family *Prevotellaceae* , unidentified species	4.276059	8.41E-07	2.31E-06	-4.950924	8.80E-08	2.42E-07	-0.674865	0.371236	0.5105
Cyanobacteria		order *Streptophyta* , unidentified species	-1.87176	0.010768	0.016921	0.33523	0.64307	0.785975	-1.536529	0.034952	0.076895
Firmicutes	*Christensenellaceae*	family *Christensenellaceae* , unidentified species	-0.022463	0.952644	0.952644	1.49498	0.000266	0.000488	1.472517	0.000224	0.000821
Firmicutes	*Lachnospiraceae*	*Roseburia* sp.	3.560056	0.000917	0.001681	-0.158486	0.882346	0.97058	3.401571	0.002115	0.005817
Firmicutes	*Peptostreptococcaceae*	family *Peptostreptococcaceae* , unidentified species	0.250238	0.701773	0.809385	-0.472333	0.508397	0.699046	-0.222095	0.748138	0.748138
Firmicutes	*Ruminococcaceae*	*Ruminococcus flavefaciens*	-5.918755	2.55E-16	2.81E-15	6.518785	2.35E-18	2.59E-17	0.60003	0.153294	0.281039
Firmicutes	*Turicibacteraceae*	*Turicibacter* sp.	0.314812	0.565817	0.777998	0.005631	0.991932	0.991932	0.320443	0.559398	0.615338
Proteobacteria	*Alcaligenaceae*	*Sutterella* sp.	-2.287873	3.77E-05	8.29E-05	2.588637	7.92E-06	1.74E-05	0.300764	0.552334	0.615338
Proteobacteria	*Enterobacteriaceae*	family *Enterobacteriaceae* , unidentified species	-4.222049	6.59E-09	2.42E-08	1.615504	0.00925	0.014536	-2.606545	4.55E-05	0.00025
			Within Jax								
			A/J relative to BALB/c			C57BL/6 relative to A/J			C57BL/6 relative to BALB/c		
**Phylum**	**Family**	**Operational taxonomic unit (OTU)**	**logFC**	**P.Value**	**adj.P.Val**	**logFC**	**P.Value**	**adj.P.Val**	**logFC**	**P.Value**	**adj.P.Val**
Actinobacteria	*Coriobacteriaceae*	family *Coriobacteriaceae* , unidentified species	0.023152	0.966975	0.969856	1.516916	0.004414	0.016186	1.540068	0.004177	0.011487
Bacteroidetes	[*Paraprevotellaceae*]	[*Prevotella*] sp.	0.351769	0.669613	0.969856	-0.961211	0.22184	0.324563	-0.609441	0.466395	0.641294
Bacteroidetes	*Prevotellaceae*	family *Prevotellaceae* , unidentified species	0.617625	0.424817	0.969856	-0.872259	0.236046	0.324563	-0.254634	0.746652	0.798125
Cyanobacteria		order *Streptophyta* , unidentified species	1.739245	0.023796	0.13088	-1.944657	0.009213	0.025335	-0.205412	0.776018	0.798125
Firmicutes	*Christensenellaceae*	family *Christensenellaceae* , unidentified species	-0.027642	0.949661	0.969856	-0.080918	0.845872	0.845872	-0.10856	0.798125	0.798125
Firmicutes	*Lachnospiraceae*	*Roseburia* sp.	-3.128044	0.003472	0.038197	-0.321682	0.761697	0.837866	-3.449726	0.001351	0.004954
Firmicutes	*Peptostreptococcaceae*	family *Peptostreptococcaceae* , unidentified species	0.162965	0.828901	0.969856	4.039208	3.75E-06	2.06E-05	4.202173	8.36E-07	4.60E-06
Firmicutes	*Ruminococcaceae*	*Ruminococcus flavefaciens*	-0.343079	0.42072	0.969856	-0.393463	0.349393	0.427036	-0.736542	0.085771	0.157247
Firmicutes	*Turicibacteraceae*	*Turicibacter* sp.	0.02311	0.969856	0.969856	9.487369	3.28E-20	3.61E-19	9.510479	5.65E-21	6.21E-20
Proteobacteria	*Alcaligenaceae*	*Sutterella* sp.	-0.061011	0.909715	0.969856	-0.800355	0.19217	0.324563	-0.861366	0.162251	0.254965
Proteobacteria	*Enterobacteriaceae*	family *Enterobacteriaceae* , unidentified species	0.491713	0.465158	0.969856	1.17525	0.059959	0.131909	1.666964	0.011236	0.024719

Table S3
