# Supplementary material for: Effects of Vendor and Genetic Background on the Composition of the Fecal Microbiota of Inbred Mice
Source: PLoS One. 2015 Feb 12;10(2):e0116704. doi: 10.1371/journal.pone.0116704 (PMC4326421; doi:10.1371/journal.pone.0116704)
Supplement: S4 Table — Pairwise comparisons within variables of the relative abundance of operational taxonomic units (OTUs) with detected interactions between strain and vendor at 10.5 weeks of age. Log-fold difference between groups (logFC), calculated p values (P.Value), and p values adjusted to control false discovery (adj.P.Val) are shown. Adjusted p values below 0.05 are shaded in grey. Genus names in square brackets are annotations supplied by the Greengenes database and not officially accepted by the Society for General Microbiology, typically due to polyphyly of the genus. (PDF) [file pone.0116704.s004.pdf]

OTUs with interactions at 10.5 weeks			Within A/J			Within BALB/c			Within C57BL/6		
			HSD relative to Jax			HSD relative to Jax			HSD relative to Jax		
**Phylum**	**Family**	**Operational taxonomic unit (OTU)**	**logFC**	**P.Value**	**adj.P.Val**	**logFC**	**P.Value**	**adj.P.Val**	**logFC**	**P.Value**	**adj.P.Val**
No BLAST hit		No BLAST hit	-0.566766	0.173541	0.280336	-0.379747	0.212487	0.247902	0.773826	0.042662	0.055994
Actinobacteria	*Coriobacteriaceae*	family *Coriobacteriaceae* , unidentified species	1.305668	0.000102	0.000306	1.473015	4.43E-08	9.30E-08	-0.147255	0.541351	0.541351
Bacteroidetes	*Bacteroidaceae*	*Bacteroides acidifaciens*	5.46379	3.20E-07	1.12E-06	8.349885	6.52E-16	3.42E-15	6.116469	2.74E-09	1.44E-08
Bacteroidetes	*Bacteroidaceae*	*Bacteroides ovatus*	5.90384	2.61E-10	1.82E-09	8.327984	2.93E-19	2.05E-18	7.966577	1.97E-15	1.38E-14
Bacteroidetes	*Bacteroidaceae*	*Bacteroides uniformis*	2.335582	0.006532	0.015242	5.44046	1.99E-11	5.96E-11	4.716341	7.84E-08	2.35E-07
Bacteroidetes	[*Paraprevotellaceae*]	[*Prevotella*] sp.	4.16264	0.018462	0.035245	-0.276786	0.832892	0.874537	-1.437368	0.395479	0.415253
Bacteroidetes	*Porphyromonadaceae*	*Parabacteroides distasonis*	0.523083	0.710359	0.785134	3.968557	0.000258	0.000362	7.267784	3.70E-07	9.72E-07
Bacteroidetes	*Porphyromonadaceae*	*Parabacteroides* sp.	0.790337	0.348975	0.488565	2.56724	0.000159	0.000238	3.999318	5.28E-06	1.11E-05
Bacteroidetes	*Prevotellaceae*	*Prevotella* sp.	5.76238	6.53E-11	6.85E-10	8.013503	9.13E-20	9.59E-19	7.637683	6.94E-16	7.29E-15
Bacteroidetes	*Rikenellaceae*	AF12 sp.	8.792692	1.08E-17	2.27E-16	7.911382	3.06E-21	6.42E-20	6.937052	6.03E-16	7.29E-15
Cyanobacteria		order YS2, unidentified species	0.89209	0.391743	0.514162	4.571092	1.05E-07	2.00E-07	3.838784	7.38E-05	0.000129
Deferribacteres	*Deferribacteraceae*	*Mucispirillum schaedleri*	7.897451	8.28E-08	4.35E-07	4.207708	2.54E-05	4.10E-05	5.910917	2.11E-06	4.93E-06
Firmicutes	*Lachnospiraceae*	*Blautia* sp.	0.589179	0.487119	0.601735	-2.019611	0.00355	0.004659	-0.968418	0.22312	0.246607
Firmicutes	*Lachnospiraceae*	*Ruminococcus flavefaciens*	0.804414	0.566813	0.661282	7.718152	6.90E-10	1.61E-09	4.340522	0.00073	0.001179
Firmicutes	*Lachnospiraceae*	*Ruminococcus* sp.	-0.218528	0.77707	0.815923	-2.727539	1.71E-05	2.99E-05	-1.669495	0.020522	0.02873
Firmicutes	*Peptococcaceae*	family *Peptococcaceae* , unidentified species	4.280934	1.81E-07	7.61E-07	4.868318	8.61E-12	3.62E-11	1.916707	0.004483	0.006725
Firmicutes	*Turicibacteraceae*	*Turicibacter* sp.	0.140307	0.928708	0.928708	0.019925	0.98532	0.98532	-8.773885	2.48E-08	8.69E-08
Proteobacteria	*Alcaligenaceae*	*Sutterella* sp.	3.156129	0.002141	0.00562	6.510895	1.88E-11	5.96E-11	6.287102	4.66E-09	1.96E-08
Proteobacteria		order RF32, unidentified species	-3.045722	0.017242	0.035245	2.003449	0.022108	0.02731	1.383108	0.190582	0.222346
Proteobacteria	*Enterobacteriaceae*	family *Enterobacteriaceae* , unidentified species	2.061003	0.051825	0.090694	6.50897	1.95E-10	5.12E-10	1.584003	0.114468	0.141401
Proteobacteria	*Halomonadaceae*	*Halomonas* sp.	-0.420989	0.26841	0.402615	-0.059056	0.789753	0.872885	0.983727	4.18E-05	7.97E-05
						**Within HSD**					
			**A/J relative to BALB/c**			**C57BL/6 relative to A/J**			**C57BL/6 relative to BALB/c**		
**Phylum**	**Family**	**Operational taxonomic unit (OTU)**	**logFC**	**P.Value**	**adj.P.Val**	**logFC**	**P.Value**	**adj.P.Val**	**logFC**	**P.Value**	**adj.P.Val**
No BLAST hit		No BLAST hit	0.041995	0.873816	0.873816	0.709054	0.011292	0.013949	0.751048	0.006997	0.02449
Actinobacteria	*Coriobacteriaceae*	family *Coriobacteriaceae* , unidentified species	-0.378007	0.057715	0.0808	0.197529	0.329819	0.34631	-0.180478	0.36456	0.54684
Bacteroidetes	*Bacteroidaceae*	*Bacteroides acidifaciens*	-1.28123	0.032178	0.05034	1.38133	0.023726	0.02768	0.1001	0.864399	0.908362
Bacteroidetes	*Bacteroidaceae*	*Bacteroides ovatus*	-1.498758	0.002318	0.006641	1.37357	0.005606	0.008409	-0.125187	0.789313	0.908362
Bacteroidetes	*Bacteroidaceae*	*Bacteroides uniformis*	-0.949086	0.084466	0.110862	1.574577	0.006237	0.008732	0.62549	0.253931	0.410196
Bacteroidetes	[*Paraprevotellaceae*]	[*Prevotella*] sp.	5.435712	2.28E-05	0.00012	-5.637552	1.76E-05	7.40E-05	-0.20184	0.865107	0.908362
Bacteroidetes	*Porphyromonadaceae*	*Parabacteroides distasonis*	-2.162666	0.018998	0.036269	6.356682	1.49E-08	3.14E-07	4.194016	2.11E-05	0.000148
Bacteroidetes	*Porphyromonadaceae*	*Parabacteroides* sp.	-0.353038	0.520967	0.607795	3.238531	6.93E-07	7.27E-06	2.885492	4.70E-06	6.87E-05
Bacteroidetes	*Prevotellaceae*	*Prevotella* sp.	-1.68154	0.000367	0.00154	1.460181	0.001942	0.003137	-0.22136	0.615129	0.776339
Bacteroidetes	*Rikenellaceae*	AF12 sp.	1.032428	0.012485	0.026219	-2.098117	5.36E-06	3.34E-05	-1.065688	0.010593	0.03178
Cyanobacteria		order YS2, unidentified species	-2.123686	0.002454	0.006641	2.561827	0.000498	0.000951	0.438141	0.49842	0.697788
Deferribacteres	*Deferribacteraceae*	*Mucispirillum schaedleri*	4.088126	5.04E-06	3.53E-05	-2.203998	0.008314	0.010912	1.884129	0.021272	0.049634
Firmicutes	*Lachnospiraceae*	*Blautia* sp.	1.286578	0.024816	0.043428	-2.509186	4.24E-05	0.000148	-1.222608	0.03791	0.079611
Firmicutes	*Lachnospiraceae*	*Ruminococcus flavefaciens*	-7.61288	1.34E-10	2.81E-09	3.561734	0.00042	0.000951	-4.051146	3.10E-05	0.000163
Firmicutes	*Lachnospiraceae*	*Ruminococcus* sp.	1.458683	0.005002	0.011672	-2.045531	0.000196	0.000515	-0.586848	0.243424	0.410196
Firmicutes	*Peptococcaceae*	family *Peptococcaceae* , unidentified species	-0.413669	0.382314	0.47227	-2.012945	0.000129	0.000388	-2.426615	6.54E-06	6.87E-05
Firmicutes	*Turicibacteraceae*	*Turicibacter* sp.	0.408807	0.669177	0.702635	-0.358565	0.715983	0.715983	0.050243	0.95741	0.95741
Proteobacteria	*Alcaligenaceae*	*Sutterella* sp.	-1.994602	0.00253	0.006641	2.299499	0.000746	0.001306	0.304897	0.628465	0.776339
Proteobacteria		order RF32, unidentified species	-1.751296	0.03356	0.05034	4.250754	6.37E-06	3.34E-05	2.499458	0.002085	0.008757
Proteobacteria	*Enterobacteriaceae*	family *Enterobacteriaceae* , unidentified species	-4.421983	1.30E-07	1.37E-06	2.687273	0.000488	0.000951	-1.734711	0.018304	0.048049
Proteobacteria	*Halomonadaceae*	*Halomonas* sp.	-0.128392	0.565955	0.625529	0.349622	0.181699	0.200825	0.22123	0.239501	0.410196
						**Within Jax**					
			**A/J relative to BALB/c**			**C57BL/6 relative to A/J**			**C57BL/6 relative to BALB/c**		
**Phylum**	**Family**	**Operational taxonomic unit (OTU)**	**logFC**	**P.Value**	**adj.P.Val**	**logFC**	**P.Value**	**adj.P.Val**	**logFC**	**P.Value**	**adj.P.Val**
No BLAST hit		No BLAST hit	0.229014	0.454551	0.650062	-0.631539	0.025019	0.105078	-0.402525	0.196309	0.34354
Actinobacteria	*Coriobacteriaceae*	family *Coriobacteriaceae* , unidentified species	-0.21066	0.359798	0.581212	1.650452	1.30E-10	1.36E-09	1.439792	1.56E-07	1.64E-06
Bacteroidetes	*Bacteroidaceae*	*Bacteroides acidifaciens*	1.604865	0.020689	0.144822	0.728651	0.229653	0.475958	2.333515	0.001254	0.004388
Bacteroidetes	*Bacteroidaceae*	*Bacteroides ovatus*	0.925386	0.089505	0.208845	-0.689167	0.158674	0.475958	0.236219	0.663286	0.773834
Bacteroidetes	*Bacteroidaceae*	*Bacteroides uniformis*	2.155792	0.005423	0.05694	-0.806182	0.231457	0.475958	1.34961	0.073083	0.153475
Bacteroidetes	[*Paraprevotellaceae*]	[*Prevotella*] sp.	0.996286	0.486149	0.650062	-0.037544	0.976895	0.97963	0.958742	0.507255	0.640383
Bacteroidetes	*Porphyromonadaceae*	*Parabacteroides distasonis*	1.282808	0.204791	0.390965	-0.38802	0.679989	0.892486	0.894788	0.379986	0.599166
Bacteroidetes	*Porphyromonadaceae*	*Parabacteroides* sp.	1.423865	0.03157	0.16574	0.029549	0.959604	0.97963	1.453414	0.030535	0.071248
Bacteroidetes	*Prevotellaceae*	*Prevotella* sp.	0.569583	0.271675	0.475431	-0.415123	0.380873	0.666527	0.15446	0.766965	0.847698
Bacteroidetes	*Rikenellaceae*	AF12 sp.	0.151118	0.765286	0.824701	-0.242476	0.572969	0.832026	-0.091358	0.856469	0.856469
Cyanobacteria		order YS2, unidentified species	1.555317	0.056551	0.169654	-0.384867	0.594304	0.832026	1.17045	0.150849	0.287984
Deferribacteres	*Deferribacteraceae*	*Mucispirillum schaedleri*	0.398384	0.664458	0.820801	-0.217464	0.801518	0.97963	0.18092	0.845401	0.856469
Firmicutes	*Lachnospiraceae*	*Blautia* sp.	-1.322212	0.046753	0.169654	-0.951589	0.096274	0.336957	-2.273802	0.001086	0.004388
Firmicutes	*Lachnospiraceae*	*Ruminococcus flavefaciens*	-0.699142	0.495286	0.650062	0.025626	0.97963	0.97963	-0.673516	0.518406	0.640383
Firmicutes	*Lachnospiraceae*	*Ruminococcus* sp.	-1.050328	0.071632	0.188033	-0.594565	0.249311	0.475958	-1.644893	0.006499	0.017059
Firmicutes	*Peptococcaceae*	family *Peptococcaceae* , unidentified species	0.173715	0.776443	0.824701	0.351282	0.548006	0.832026	0.524996	0.399444	0.599166
Firmicutes	*Turicibacteraceae*	*Turicibacter* sp.	0.288426	0.785429	0.824701	8.555627	2.27E-12	4.76E-11	8.844053	1.76E-10	3.70E-09
Proteobacteria	*Alcaligenaceae*	*Sutterella* sp.	1.360164	0.055599	0.169654	-0.831474	0.229419	0.475958	0.52869	0.457665	0.640383
Proteobacteria		order RF32, unidentified species	3.297874	0.001563	0.032827	-0.178076	0.84247	0.97963	3.119799	0.002904	0.008711
Proteobacteria	*Enterobacteriaceae*	family *Enterobacteriaceae* , unidentified species	0.025984	0.976756	0.976756	3.164272	0.000349	0.001831	3.190256	0.000993	0.004388
Proteobacteria	*Halomonadaceae*	*Halomonas* sp.	0.233541	0.200125	0.390965	-1.055093	1.98E-07	1.39E-06	-0.821553	5.06E-05	0.000354

Table S4
