# Supplementary material for: Effects of Vendor and Genetic Background on the Composition of the Fecal Microbiota of Inbred Mice
Source: PLoS One. 2015 Feb 12;10(2):e0116704. doi: 10.1371/journal.pone.0116704 (PMC4326421; doi:10.1371/journal.pone.0116704)
Supplement: S5 Table — Testing for vendor- and strain-dependent main effects on the relative abundance of phyla and operational taxonomic units (OTUs) with no interactions between variables in 3.5 week old A/J, BALB/c, and C57BL/6 mice purchased from Harlan Laboratories (HSD) and The Jackson Laboratory (Jax). Log-normalized average abundance (AveExpr) of each OTU (averaged across all samples), log2 fold difference between groups (logFC), calculated p values (P.Value), and adjusted p values (adj.P.Val) are shown. Adjusted p values below 0.05 are shaded in grey. Taxon names above the rank of genus in square brackets are names proposed by the Greengenes curators and will not be found in NCBI. Genus names in square brackets are annotations supplied by the Greengenes database and not officially accepted by the Society for General Microbiology, typically due to polyphyly of the genus. (PDF) [file pone.0116704.s005.pdf]

Phyla without vendor × strain interactions at 3.5 weeks	Main Effects - Vendor (Jax relative to HSD)						Main Effects - Strain			A/J relative to BALB/c			C57BL/6 relative to A/J			C57BL/6 relative to BALB/c		
Phylum	logFC	AveExpr	t	P.Value	adj.P.Val	B	F	P.Value	adj.P.Val	logFC	P.Value	adj.P.Val	logFC	P.Value	adj.P.Val	logFC	P.Value	adj.P.Val
Actinobacteria	-0.12098	4.072324	-0.18661	0.852772	0.852772	-6.57223	2.752666	0.07412	0.444722	-0.82841	0.116497	0.450108	2.173125	2.11E-05	0.000127	1.344712	0.011646	0.069876
Bacteroidetes	-0.52225	14.85796	-1.15037	0.255852	0.511703	-5.97738	0.887856	0.418372	0.998528	-0.27687	0.450108	0.450108	0.090781	0.773842	0.84045	-0.18609	0.611056	0.785559
Cyanobacteria	-0.41212	3.587458	-0.61543	0.541263	0.649516	-6.41005	0.120892	0.886406	0.998528	0.569389	0.31465	0.450108	-0.44169	0.421362	0.84045	0.127697	0.833767	0.833767
Firmicutes	-0.29815	16.13947	-0.68943	0.493963	0.649516	-6.39494	0.074317	0.928487	0.998528	-0.27312	0.436325	0.450108	0.116533	0.699972	0.84045	-0.15658	0.654632	0.785559
Tenericutes	1.361922	9.04063	2.34328	0.023428	0.070284	-4.02872	0.001473	0.998528	0.998528	0.349172	0.448944	0.450108	-1.10096	0.007548	0.022643	-0.75179	0.106554	0.319662
TM7	-2.48252	2.401074	-3.51868	0.000979	0.005874	-0.98322	0.16926	0.844805	0.998528	-0.68753	0.219351	0.450108	0.101043	0.84045	0.84045	-0.58649	0.284373	0.568745

	OTUs without vendor × strain interactions at 3.5 weeks		Main Effects - Vendor (Jax relative to HSD)						Main Effects - Strain			A/J relative to BALB/c			C57BL/6 relative to A/J			C57BL/6 relative to BALB/c		
Phylum	Family	Operational taxonomic unit (OTU)	logFC	AveExpr	t	P.Value	adj.P.Val	B	F	P.Value	adj.P.Val	logFC	P.Value	adj.P.Val	logFC	P.Value	adj.P.Val	logFC	P.Value	adj.P.Val
No BLAST hit		No BLAST hit	-1.64009	1.594889	-1.91699	0.062467	0.098162	-4.23344	7.751032	0.001442	0.012685	-0.77052	0.219696	0.634229	0.52583	0.355407	0.600857	-0.24469	0.707226	0.946865
Actinobacteria	*Coriobacteriaceae*	*Adlercreutzia* sp.	0.586012	3.129167	0.789551	0.434487	0.525521	-5.97365	0.708507	0.498507	0.722658	0.808598	0.154437	0.554552	-0.89308	0.082587	0.302817	-0.08448	0.882306	0.946865
Bacteroidetes	[*Odoribacteraceae*]	*Odoribacter* sp.	-5.92579	3.879872	-3.41168	0.001499	0.00347	-1.23138	0.20838	0.812782	0.927796	-0.63208	0.617155	0.805315	0.164443	0.893705	0.893705	-0.46763	0.738023	0.946865
Bacteroidetes	*Bacteroidaceae*	*Bacteroides acidifaciens*	-7.92188	8.039285	-9.04394	3.50E-11	7.71E-10	15.16478	1.786562	0.180762	0.361523	-0.45623	0.42518	0.805315	0.386035	0.459283	0.661466	-0.0702	0.906549	0.949718
Bacteroidetes	*Bacteroidaceae*	*Bacteroides ovatus*	-4.79265	3.81991	-5.61742	1.67E-06	1.47E-05	4.931255	8.506237	0.000844	0.012375	-1.19906	0.020464	0.261201	-0.52228	0.309037	0.570608	-1.72134	0.004665	0.051311
Bacteroidetes	*Bacteroidaceae*	*Bacteroides uniformis*	-4.90963	3.444278	-3.91537	0.000346	0.001088	0.177294	0.532411	0.591327	0.765247	-0.58985	0.439705	0.805315	-0.41524	0.566652	0.692574	-1.00509	0.224513	0.757549
Bacteroidetes	*Bacteroidaceae*	*Bacteroides* sp.	-7.76009	6.441957	-10.237	1.09E-12	4.78E-11	18.30429	0.113104	0.893345	0.942794	0.330794	0.51522	0.805315	-0.7316	0.131974	0.446682	-0.40081	0.465837	0.854035
Bacteroidetes	*Porphyromonadaceae*	*Parabacteroides distasonis*	-6.18166	4.136032	-5.03371	1.09E-05	5.97E-05	3.31329	5.540816	0.007552	0.03323	0.153118	0.846037	0.900486	0.868729	0.233433	0.553009	1.021847	0.21744	0.757549
Bacteroidetes	*Porphyromonadaceae*	*Parabacteroides* sp.	-3.38426	2.793971	-2.78585	0.00815	0.017929	-2.59775	0.190994	0.826893	0.927796	0.255265	0.725402	0.870654	-0.41396	0.548583	0.692574	-0.15869	0.839811	0.946865
Bacteroidetes	*Prevotellaceae*	*Prevotella* sp.	-4.67126	3.918466	-5.91691	6.38E-07	7.02E-06	5.912902	6.114045	0.004849	0.025147	-0.98824	0.06498	0.345834	0.32244	0.517019	0.689358	-0.6658	0.241038	0.757549
Bacteroidetes	*Rikenellaceae*	AF12 sp.	-5.43277	4.011584	-5.32056	4.34E-06	2.73E-05	4.171968	3.116626	0.055322	0.128115	-0.79337	0.278208	0.720069	-0.70697	0.322487	0.570608	-1.50034	0.06745	0.370974
Bacteroidetes	*Rikenellaceae*	family *Rikenellaceae* , unidentified species	2.151736	10.84666	1.004783	0.321096	0.415535	-5.8424	0.64083	0.532225	0.722658	-1.72442	0.214069	0.634229	-1.39775	0.268689	0.570608	-3.12218	0.035239	0.258419
Cyanobacteria		order *Streptophyta* , unidentified species	-1.87075	2.82565	-2.0952	0.042589	0.069405	-4.03345	7.084221	0.002342	0.015611	-0.92802	0.070739	0.345834	-1.36488	0.008532	0.062569	-2.2929	0.000203	0.00892
Cyanobacteria		order YS2, unidentified species	-2.89275	1.82488	-2.09685	0.042434	0.069405	-3.83695	4.633452	0.015549	0.054876	-1.37824	0.114957	0.459826	0.80026	0.307617	0.570608	-0.57798	0.520177	0.8803
Firmicutes	[*Mogibacteriaceae*]	family [*Mogibacteriaceae*], unidentified species	3.909356	5.39149	4.938844	1.47E-05	7.15E-05	3.027059	0.622064	0.541993	0.722658	0.150016	0.771716	0.870654	-0.60429	0.204444	0.553009	-0.45427	0.404193	0.822992
Firmicutes	*Christensenellaceae*	family *Christensenellaceae* , unidentified species	-4.17007	1.684894	-3.48872	0.001204	0.002943	-0.91534	0.635703	0.534876	0.722658	0.560764	0.496558	0.805315	-0.30377	0.676918	0.726449	0.256993	0.760432	0.946865
Firmicutes	*Clostridiaceae*	Candidatus *Arthromitus*	-5.41621	3.341778	-7.47198	4.40E-09	6.45E-08	10.1647	4.425457	0.018417	0.054876	-0.82545	0.08273	0.364012	0.432223	0.306455	0.570608	-0.39322	0.411496	0.822992
Firmicutes	*Clostridiaceae*	*Clostridium* sp.	6.285013	3.531087	4.836592	2.03E-05	8.11E-05	2.743045	3.973226	0.026748	0.06923	-0.54363	0.517	0.805315	1.390944	0.059801	0.29236	0.847314	0.305254	0.769782
Firmicutes	*Clostridiaceae*	family *Clostridiaceae* , unidentified species	2.748873	4.125705	2.426348	0.019896	0.038062	-3.462	9.870261	0.000332	0.007308	-2.05903	0.004173	0.091803	2.051067	0.00159	0.017493	-0.00796	0.99103	0.99103
Firmicutes		order *Clostridiales* , unidentified species	0.918824	14.60768	1.435445	0.159005	0.225684	-5.34024	0.082066	0.921367	0.942794	0.240219	0.570095	0.805315	-0.45759	0.238799	0.553009	-0.21737	0.624129	0.946865
Firmicutes	*Dehalobacteriaceae*	*Dehalobacterium* sp.	-0.51987	5.767456	-0.717	0.47758	0.552987	-6.08384	0.102159	0.903123	0.942794	-0.13856	0.770683	0.870654	-0.15791	0.715851	0.749939	-0.29648	0.552909	0.890491
Firmicutes	*Erysipelotrichaceae*	*Coprobacillus* sp.	1.123162	2.324698	1.224383	0.228035	0.313548	-5.57795	1.821255	0.175104	0.361523	0.793837	0.230629	0.634229	-0.35102	0.551585	0.692574	0.442822	0.515428	0.8803
Firmicutes	*Erysipelotrichaceae*	family *Erysipelotrichaceae* , unidentified species	2.689451	6.095033	3.752933	0.00056	0.001643	-0.37959	1.909016	0.161607	0.355536	-0.08176	0.863614	0.900486	-1.55269	0.000752	0.013934	-1.63444	0.001872	0.027457
Firmicutes	*Lachnospiraceae*	*Anaerostipes* sp.	3.972292	6.517977	2.274632	0.028414	0.052093	-3.8991	0.044799	0.956238	0.956238	-0.9321	0.415074	0.805315	0.988132	0.324209	0.570608	0.056033	0.961466	0.983826
Firmicutes	*Lachnospiraceae*	*Blautia* sp.	-0.48128	2.151563	-0.38812	0.700007	0.770008	-6.08174	4.715891	0.014545	0.054876	1.279628	0.163845	0.554552	-1.50303	0.078359	0.302817	-0.2234	0.816756	0.946865
Firmicutes	*Lachnospiraceae*	*Coprococcus* sp.	2.058689	7.450378	2.444518	0.019049	0.038062	-3.5781	0.238697	0.788778	0.927796	0.288562	0.599514	0.805315	-1.14636	0.026341	0.144875	-0.8578	0.141461	0.691586
Firmicutes	*Lachnospiraceae*	*Dorea* sp.	-0.11312	3.766836	-0.12388	0.902033	0.902033	-6.29312	0.201795	0.818097	0.927796	1.433616	0.023746	0.261201	-0.75397	0.20468	0.553009	0.679649	0.314911	0.769782
Firmicutes	*Lachnospiraceae*	*Roseburia* sp.	-2.4188	0.741497	-1.76561	0.085163	0.129212	-4.46206	3.670497	0.034476	0.084274	2.086474	0.044399	0.325589	-1.26248	0.171792	0.539919	0.823995	0.434412	0.83105
Firmicutes	*Lachnospiraceae*	*Ruminococcus gnavus*	0.884545	7.583041	1.049219	0.300433	0.400577	-5.79826	4.406305	0.018708	0.054876	0.56721	0.304812	0.745096	-1.77379	0.00095	0.013934	-1.20658	0.041305	0.25963
Firmicutes	*Lachnospiraceae*	family *Lachnospiraceae* , unidentified species	1.614003	12.11767	2.122948	0.040054	0.069405	-4.22015	1.154585	0.32556	0.530543	-0.07505	0.880021	0.900486	-1.32024	0.00554	0.048751	-1.3953	0.010263	0.090317
Firmicutes	*Peptococcaceae*	family *Peptococcaceae* , unidentified species	-4.05961	1.632833	-4.41995	7.46E-05	0.000274	1.538151	0.750694	0.47863	0.722658	0.369021	0.531749	0.805315	-0.26413	0.624909	0.707985	0.104891	0.864374	0.946865
Firmicutes	*Ruminococcaceae*	*Anaerotruncus* sp.	-0.35288	0.605095	-0.50854	0.613895	0.6926	-5.88874	8.096679	0.001126	0.012387	2.160099	0.0013	0.057207	-2.0285	0.000923	0.013934	0.131601	0.840884	0.946865
Firmicutes	*Ruminococcaceae*	*Oscillospira* sp.	0.597043	11.79442	0.776752	0.441916	0.525521	-6.04012	0.170986	0.843451	0.927796	-0.3413	0.498157	0.805315	-0.38199	0.406629	0.638989	-0.72329	0.174947	0.736356
Firmicutes	*Ruminococcaceae*	*Ruminococcus* sp.	0.828589	9.776695	0.823007	0.415428	0.522252	-6.00395	1.161327	0.323493	0.530543	-0.61012	0.352311	0.805315	-0.30921	0.604041	0.707985	-0.91933	0.184089	0.736356
Firmicutes	*Ruminococcaceae*	family *Ruminococcaceae* , unidentified species	1.03595	11.40883	1.685086	0.09983	0.146418	-4.97636	0.258917	0.773182	0.927796	-0.19	0.640592	0.805315	-0.27738	0.455951	0.661466	-0.46739	0.276592	0.769782
Proteobacteria	*Alcaligenaceae*	*Sutterella* sp.	-4.76145	3.724457	-5.40729	3.29E-06	2.41E-05	4.384576	6.037082	0.005144	0.025147	-0.14185	0.821856	0.900486	0.399403	0.496993	0.683366	0.257554	0.698802	0.946865
Proteobacteria	*Desulfovibrionaceae*	*Bilophila* sp.	-5.01071	3.004966	-4.90658	1.62E-05	7.15E-05	2.934928	1.700108	0.195712	0.374406	0.224942	0.744822	0.870654	0.443624	0.466033	0.661466	0.668566	0.340779	0.789172
Proteobacteria	*Desulfovibrionaceae*	*Desulfovibrio* C21_c20	-0.14262	1.158877	-0.133	0.894863	0.902033	-5.93876	13.44816	3.48E-05	0.001533	-1.5745	0.041	0.325589	1.689745	0.013245	0.083257	0.115242	0.878289	0.946865
Proteobacteria	*Desulfovibrionaceae*	*Desulfovibrio* sp.	-4.50955	2.950575	-3.73008	0.000599	0.001647	-0.30761	1.276187	0.290317	0.510957	0.509736	0.506527	0.805315	0.290577	0.662226	0.726449	0.800313	0.296064	0.769782
Proteobacteria	*Enterobacteriaceae*	family *Enterobacteriaceae* , unidentified species	-0.33068	1.13025	-0.24046	0.811212	0.849841	-5.81161	7.004541	0.002484	0.015611	-0.46949	0.606878	0.805315	1.051269	0.238179	0.553009	0.581782	0.566676	0.890491
Proteobacteria	*Halomonadaceae*	*Halomonas* sp.	-0.20576	0.419747	-0.26436	0.792873	0.849841	-5.74314	1.035705	0.364383	0.572601	0.017489	0.972175	0.972175	0.078141	0.84928	0.869031	0.09563	0.846629	0.946865
Proteobacteria	*mitochondria*	*Zea luxurians*	-2.81394	3.184449	-2.72215	0.009591	0.020095	-2.77773	4.226254	0.021689	0.059644	-1.13623	0.058635	0.345834	-1.04105	0.068193	0.300047	-2.17728	0.001443	0.027457
Tenericutes		order RF39, unidentified species	5.210934	8.446744	4.133453	0.00018	0.000608	0.68058	1.276281	0.290291	0.510957	-0.41203	0.614077	0.805315	0.676785	0.368708	0.600857	0.264757	0.758257	0.946865
TM7	F16	family F16, unidentified species	-4.46961	2.884249	-3.53895	0.001043	0.002699	-0.83829	4.467526	0.017795	0.054876	0.412253	0.63221	0.805315	0.407283	0.627532	0.707985	0.819536	0.389182	0.822992

Table S5
