# Supplementary material for: Effects of Vendor and Genetic Background on the Composition of the Fecal Microbiota of Inbred Mice
Source: PLoS One. 2015 Feb 12;10(2):e0116704. doi: 10.1371/journal.pone.0116704 (PMC4326421; doi:10.1371/journal.pone.0116704)
Supplement: S7 Table — Testing for vendor- and strain-dependent main effects on the relative abundance of phyla and operational taxonomic units (OTUs) with no interactions between variables in 10.5 week old A/J, BALB/c, and C57BL/6 mice purchased from Harlan Laboratories (HSD) and The Jackson Laboratory (Jax). Log-normalized average abundance (AveExpr) of each OTU (averaged across all samples), log2 fold difference between groups (logFC), calculated p values (P.Value), and adjusted p values (adj.P.Val) are shown. Adjusted p values below 0.05 are shaded in grey. Taxon names above the rank of genus in square brackets are names proposed by the Greengenes curators and will not be found in NCBI. Genus names in square brackets are annotations supplied by the Greengenes database and not officially accepted by the Society for General Microbiology, typically due to polyphyly of the genus. (PDF) [file pone.0116704.s007.pdf]

| Phyla without vendor × strain interactions at 10.5 weeks | Main Effects - Vendor (Jax relative to HSD) | | | | | | Main Effects - Strain | | | A/J relative to BALB/c | | | C57BL/6 relative to A/J | | | C57BL/6 relative to BALB/c | | |
|---|---|---|---|---|---|---|---|---|---|---|---|---|---|---|---|---|---|---|
| **Phylum** | **logFC** | **AveExpr** | **t** | **P.Value** | **adj.P.Val** | **B** | **F** | **P.Value** | **adj.P.Val** | **logFC** | **P.Value** | **adj.P.Val** | **logFC** | **P.Value** | **adj.P.Val** | **logFC** | **P.Value** | **adj.P.Val** |
| *Actinobacteria* | -1.75115 | 3.828834 | -2.59487 | 0.012795 | 0.02559 | -3.42723 | 0.360801 | 0.699153 | 0.838984 | -0.6921 | 0.091869 | 0.243503 | 0.572413 | 0.172466 | 0.258699 | -0.11968 | 0.766109 | 0.91933 |
| *Bacteroidetes* | -0.76445 | 15.86662 | -1.91755 | 0.061649 | 0.075557 | -4.97581 | 0.573073 | 0.567926 | 0.838984 | -0.43389 | 0.121752 | 0.243503 | 0.43371 | 0.129059 | 0.258118 | -0.00018 | 0.999479 | 0.999479 |
| *Firmicutes* | 0.634079 | 15.48143 | 1.907558 | 0.062964 | 0.075557 | -4.9936 | 11.66042 | 8.59E-05 | 0.000515 | 0.546473 | 0.021776 | 0.130655 | -1.4354 | 1.88E-07 | 1.13E-06 | -0.88892 | 0.000414 | 0.002484 |
| *Proteobacteria* | -4.3955 | 7.122821 | -6.55866 | 5E-08 | 1.5E-07 | 8.311114 | 0.672761 | 0.515449 | 0.838984 | -0.50171 | 0.294398 | 0.307276 | -0.29513 | 0.5415 | 0.6498 | -0.79684 | 0.101279 | 0.202558 |
| *Tenericutes* | 0.591204 | 10.94141 | 1.508009 | 0.138673 | 0.138673 | -5.63842 | 5.914661 | 0.005297 | 0.015892 | 0.293723 | 0.283596 | 0.307276 | -0.91176 | 0.001849 | 0.005547 | -0.61804 | 0.029311 | 0.087933 |
| TM7 | -6.21207 | 3.634296 | -11.8976 | 2.28E-15 | 1.37E-14 | 23.83373 | 0.143004 | 0.86715 | 0.86715 | 0.369327 | 0.307276 | 0.307276 | 0.103817 | 0.759558 | 0.759558 | 0.473144 | 0.196308 | 0.294462 |

| | | OTUs without vendor × strain interactions at 10.5 weeks | Main Effects - Vendor (Jax relative to HSD) | | | | | | Main Effects - Strain | | | A/J relative to BALB/c | | | C57BL/6 relative to A/J | | | C57BL/6 relative to BALB/c | | |
|---|---|---|---|---|---|---|---|---|---|---|---|---|---|---|---|---|---|---|---|---|
| **Phylum** | **Family** | **Operational taxonomic unit (OTU)** | **logFC** | **AveExpr** | **t** | **P.Value** | **adj.P.Val** | **B** | **F** | **P.Value** | **adj.P.Val** | **logFC** | **P.Value** | **adj.P.Val** | **logFC** | **P.Value** | **adj.P.Val** | **logFC** | **P.Value** | **adj.P.Val** |
| *Actinobacteria* | *Coriobacteriaceae* | *Adlercreutzia* sp. | 0.248643 | 3.532529 | 0.279498 | 0.781217 | 0.806418 | -6.18658 | 0.464545 | 0.631571 | 0.777318 | -0.34662 | 0.368586 | 0.684525 | -0.15896 | 0.674028 | 0.727467 | -0.50558 | 0.215238 | 0.286983 |
| *Bacteroidetes* | [*Odoribacteraceae* ] | *Odoribacter* sp. | -10.4263 | 5.921834 | -9.78419 | 1.87E-12 | 5.98E-11 | 17.94015 | 5.384304 | 0.008233 | 0.026345 | 0.105763 | 0.83963 | 0.95129 | -1.3902 | 0.008826 | 0.028243 | -1.28444 | 0.024 | 0.059078 |
| *Bacteroidetes* | *Bacteroidaceae* | *Bacteroides* sp. | -7.29054 | 7.460373 | -8.97608 | 2.27E-11 | 3.63E-10 | 15.622 | 0.179823 | 0.83605 | 0.863019 | 0.475047 | 0.237725 | 0.633577 | -0.38629 | 0.316915 | 0.440926 | 0.088754 | 0.833408 | 0.869838 |
| *Bacteroidetes* | *Prevotellaceae* | family *Prevotellaceae*, unidentified species | 0.694929 | 0.710874 | 0.744023 | 0.460953 | 0.587245 | -5.68926 | 16.65707 | 4.54E-06 | 0.000145 | 3.130934 | 1.54E-07 | 4.92E-06 | -1.79989 | 0.000206 | 0.001318 | 1.33104 | 0.016207 | 0.047148 |
| *Bacteroidetes* | *Rikenellaceae* | family *Rikenellaceae* , unidentified species | -3.11937 | 11.47911 | -1.18201 | 0.243768 | 0.371455 | -5.69656 | 0.397219 | 0.674654 | 0.79959 | -0.14372 | 0.909535 | 0.95129 | -1.12492 | 0.357277 | 0.47637 | -1.26863 | 0.345089 | 0.441714 |
| *Bacteroidetes* | S24-7 | family 24-7, unidentified species | 1.446071 | 15.169 | 1.9484 | 0.057984 | 0.142729 | -4.57368 | 4.115166 | 0.023238 | 0.057201 | -0.28192 | 0.443497 | 0.703671 | 0.891859 | 0.014546 | 0.037242 | 0.609943 | 0.12063 | 0.200597 |
| *Bacteroidetes* | | order *Bacteroidales* , unidentified species | -3.84192 | 9.414793 | -2.15037 | 0.037254 | 0.108374 | -4.20007 | 0.334239 | 0.717746 | 0.81486 | 0.052471 | 0.95129 | 0.95129 | 0.551477 | 0.505377 | 0.646882 | 0.603948 | 0.506978 | 0.618264 |
| *Cyanobacteria* | | order *Streptophyta* , unidentified species | 1.236951 | 3.544789 | 0.890143 | 0.378396 | 0.504528 | -5.94191 | 3.332808 | 0.045224 | 0.096479 | 0.335884 | 0.62535 | 0.833731 | -2.32747 | 0.000926 | 0.004263 | -1.99158 | 0.009141 | 0.036563 |
| *Firmicutes* | [*Mogibacteriaceae* ] | family [*Mogibacteriaceae* ], unidentified species | 0.552591 | 5.395715 | 0.717256 | 0.477137 | 0.587245 | -6.12565 | 0.467724 | 0.62961 | 0.777318 | -0.33128 | 0.385045 | 0.684525 | -0.51896 | 0.159934 | 0.269363 | -0.85024 | 0.038773 | 0.085803 |
| *Firmicutes* | *Christensenellaceae* | family *Christensenellaceae* , unidentified species | -1.83636 | 1.578031 | -2.8584 | 0.006567 | 0.026269 | -2.52881 | 11.03308 | 0.000138 | 0.000786 | 0.660213 | 0.031958 | 0.189172 | 0.15595 | 0.619845 | 0.727467 | 0.816163 | 0.012807 | 0.044757 |
| *Firmicutes* | *Clostridiaceae* | Candidatus *Arthromitus* | -4.18751 | 3.246741 | -4.33507 | 8.76E-05 | 0.000467 | 1.379189 | 2.218166 | 0.121242 | 0.215542 | 0.93018 | 0.051793 | 0.23677 | 0.468766 | 0.303095 | 0.440926 | 1.398945 | 0.006795 | 0.036238 |
| *Firmicutes* | *Clostridiaceae* | *Clostridium* sp. | 1.951077 | 2.84881 | 1.675316 | 0.101209 | 0.231335 | -4.81573 | 2.551074 | 0.089877 | 0.179754 | -0.2111 | 0.682321 | 0.839779 | 1.065453 | 0.031808 | 0.063617 | 0.85435 | 0.125373 | 0.200597 |
| *Firmicutes* | *Clostridiaceae* | family *Clostridiaceae* , unidentified species | 2.265426 | 4.044229 | 2.302089 | 0.026291 | 0.093478 | -3.85901 | 1.287279 | 0.286554 | 0.436654 | -0.47857 | 0.381849 | 0.684525 | 1.701841 | 0.000933 | 0.004263 | 1.223272 | 0.04022 | 0.085803 |
| *Firmicutes* | | order *Clostridiales* , unidentified species | 0.546132 | 14.75756 | 1.040497 | 0.303987 | 0.442163 | -5.84842 | 11.23084 | 0.000121 | 0.000786 | 0.577072 | 0.03547 | 0.189172 | -1.40079 | 1.98E-06 | 2.11E-05 | -0.82372 | 0.005356 | 0.034278 |
| *Firmicutes* | *Dehalobacteriaceae* | *Dehalobacterium* sp. | 0.289033 | 6.608993 | 0.412403 | 0.682115 | 0.752678 | -6.29552 | 0.050718 | 0.950604 | 0.950604 | 0.321245 | 0.3577 | 0.684525 | -0.84019 | 0.01513 | 0.037242 | -0.51895 | 0.162486 | 0.247597 |
| *Firmicutes* | *Erysipelotrichaceae* | *Coprobacillus* sp. | 2.475808 | 2.608097 | 1.996206 | 0.052338 | 0.139569 | -4.34667 | 3.981285 | 0.026004 | 0.059439 | -1.26757 | 0.032275 | 0.189172 | -0.21799 | 0.704734 | 0.727467 | -1.48556 | 0.021366 | 0.056976 |
| *Firmicutes* | *Erysipelotrichaceae* | family *Erysipelotrichaceae* , unidentified species | 1.826576 | 6.847133 | 1.438013 | 0.157747 | 0.280439 | -5.37639 | 0.30535 | 0.738467 | 0.81486 | 0.480827 | 0.435279 | 0.703671 | -0.60971 | 0.304754 | 0.440926 | -0.12888 | 0.842655 | 0.869838 |
| *Firmicutes* | *Lachnospiraceae* | *Coprococcus* sp. | 1.214729 | 7.981926 | 1.44579 | 0.155564 | 0.280439 | -5.36577 | 4.53058 | 0.016454 | 0.046159 | -0.64997 | 0.119901 | 0.409957 | -0.70899 | 0.078835 | 0.140151 | -1.35896 | 0.003042 | 0.024338 |
| *Firmicutes* | *Lachnospiraceae* | *Dorea* sp. | 1.494374 | 4.835648 | 1.469879 | 0.148952 | 0.280439 | -5.33255 | 0.545411 | 0.583602 | 0.777318 | -0.14493 | 0.769646 | 0.912173 | 0.299927 | 0.529255 | 0.65139 | 0.154993 | 0.767016 | 0.846363 |
| *Firmicutes* | *Lachnospiraceae* | *Anaerostipes* sp. | 4.70766 | 6.899038 | 2.21905 | 0.03187 | 0.101984 | -4.06632 | 0.653766 | 0.525229 | 0.76397 | -0.75164 | 0.461784 | 0.703671 | 0.060393 | 0.950811 | 0.950811 | -0.69124 | 0.52166 | 0.618264 |
| *Firmicutes* | *Lachnospiraceae* | *Ruminococcus gnavus* | 0.643463 | 8.233669 | 0.98723 | 0.329112 | 0.457895 | -5.90084 | 0.566643 | 0.571651 | 0.777318 | -0.15643 | 0.630772 | 0.833731 | -0.71808 | 0.025602 | 0.054617 | -0.87452 | 0.013986 | 0.044757 |
| *Firmicutes* | *Lachnospiraceae* | family *Lachnospiraceae* , unidentified species | 0.980838 | 12.38196 | 1.355813 | 0.182313 | 0.307054 | -5.48545 | 11.03464 | 0.000138 | 0.000786 | 0.774959 | 0.034893 | 0.189172 | -2.25519 | 4.64E-08 | 1.49E-06 | -1.48023 | 0.000294 | 0.007186 |
| *Firmicutes* | *Lactobacillaceae* | *Lactobacillus* sp. | -0.19601 | 6.916501 | -0.12324 | 0.902497 | 0.902497 | -6.37238 | 1.764362 | 0.1836 | 0.309222 | 0.669269 | 0.38422 | 0.684525 | -0.2855 | 0.698451 | 0.727467 | 0.383767 | 0.635847 | 0.726683 |
| *Firmicutes* | *Ruminococcaceae* | *Oscillospira* sp. | 0.383114 | 12.12834 | 0.592015 | 0.556974 | 0.636542 | -6.20636 | 11.12406 | 0.00013 | 0.000786 | 0.558762 | 0.088814 | 0.355257 | -1.82634 | 4.53E-07 | 7.24E-06 | -1.26757 | 0.000538 | 0.007186 |
| *Firmicutes* | *Ruminococcaceae,* | family *Ruminococcaceae* , unidentified species | 0.467971 | 11.38596 | 0.680109 | 0.50012 | 0.592735 | -6.15116 | 4.469116 | 0.01731 | 0.046159 | 0.389871 | 0.257391 | 0.633577 | -1.38832 | 0.00011 | 0.000879 | -0.99845 | 0.007969 | 0.036432 |
| *Proteobacteria* | *Desulfovibrionaceae* | *Bilophila* sp. | -6.52845 | 2.924476 | -6.04786 | 3.24E-07 | 2.59E-06 | 6.543391 | 6.872068 | 0.002593 | 0.010372 | 0.217177 | 0.651353 | 0.833731 | -1.26624 | 0.012275 | 0.03571 | -1.04907 | 0.043352 | 0.086704 |
| *Proteobacteria* | *Desulfovibrionaceae* | *Desulfovibrio* C21_c20 | -7.83177 | 3.237614 | -5.70305 | 1.02E-06 | 6.53E-06 | 5.502363 | 1.5926 | 0.215285 | 0.344456 | -0.05352 | 0.929148 | 0.95129 | -0.81874 | 0.198988 | 0.31838 | -0.87226 | 0.17024 | 0.247622 |
| *Proteobacteria* | *Desulfovibrionaceae* | *Desulfovibrio* sp. | -3.85234 | 1.82758 | -3.52476 | 0.001029 | 0.004702 | -0.81969 | 10.93411 | 0.000147 | 0.000786 | 0.070582 | 0.892418 | 0.95129 | -1.11127 | 0.03599 | 0.067746 | -1.04069 | 0.065444 | 0.123189 |
| *Proteobacteria* | *mitochondria* | *Zea luxurians* | 1.631742 | 3.892916 | 1.20526 | 0.234765 | 0.371455 | -5.6684 | 7.016719 | 0.002325 | 0.010372 | -0.37084 | 0.574081 | 0.833731 | -2.15482 | 0.001351 | 0.005405 | -2.52566 | 0.000674 | 0.007186 |
| *Tenericutes* | *Anaeroplasmataceae* | *Anaeroplasma* sp. | 0.538365 | 10.0316 | 0.321618 | 0.749317 | 0.799271 | -6.32855 | 6.503403 | 0.003433 | 0.012205 | 1.849103 | 0.025716 | 0.189172 | -1.8603 | 0.019924 | 0.045541 | -0.01119 | 0.989509 | 0.989509 |
| *Tenericutes* | | order RF39, unidentified species | 2.208501 | 7.944629 | 1.479713 | 0.146318 | 0.280439 | -5.31884 | 2.340811 | 0.108529 | 0.20429 | -1.1093 | 0.128112 | 0.409957 | 2.066758 | 0.004376 | 0.01556 | 0.957458 | 0.212032 | 0.286983 |
| TM7 | F16 | family F16, unidentified species | -6.05556 | 3.634296 | -8.29677 | 1.96E-10 | 2.09E-09 | 13.26683 | 0.228123 | 0.796994 | 0.850127 | 0.485697 | 0.16693 | 0.485615 | 0.134125 | 0.678715 | 0.727467 | 0.619823 | 0.09955 | 0.176977 |

**Table S7**
