# Supplementary material for: Effects of Vendor and Genetic Background on the Composition of the Fecal Microbiota of Inbred Mice
Source: PLoS One. 2015 Feb 12;10(2):e0116704. doi: 10.1371/journal.pone.0116704 (PMC4326421; doi:10.1371/journal.pone.0116704)
Supplement: S8 Table — Testing for vendor- and strain-dependent main effects on the relative abundance of phyla and operational taxonomic units (OTUs) with no interactions between variables in 24 week old A/J, BALB/c, and C57BL/6 mice purchased from Harlan Laboratories (HSD) and The Jackson Laboratory (Jax). Log-normalized average abundance (AveExpr) of each OTU (averaged across all samples), log2 fold difference between groups (logFC), calculated p values (P.Value), and adjusted p values (adj.P.Val) are shown. Adjusted p values below 0.05 are shaded in grey. Taxon names above the rank of genus in square brackets are names proposed by the Greengenes curators and will not be found in NCBI. Genus names in square brackets are annotations supplied by the Greengenes database and not officially accepted by the Society for General Microbiology, typically due to polyphyly of the genus. (PDF) [file pone.0116704.s008.pdf]

Phyla without vendor × strain interactions at 24 weeks	Main Effects - Vendor (Jax relative to HSD)						Main Effects - Strain			A/J relative to BALB/c			C57BL/6 relative to A/J			C57BL/6 relative to BALB/c		
**Phylum**	**logFC**	**AveExpr**	**t**	**P.Value**	**adj.P.Val**	**B**	**F**	**P.Value**	**adj.P.Val**	**logFC**	**P.Value**	**adj.P.Val**	**logFC**	**P.Value**	**adj.P.Val**	**logFC**	**P.Value**	**adj.P.Val**
Actinobacteria	1.201633	1.729835	2.452069	0.018597	0.13018	-3.1425	1.391063	0.260421	0.583723	-0.0538	0.871088	0.871088	-0.2437	0.511697	0.59698	-0.2975	0.402322	0.676387
Bacteroidetes	0.374311	13.72449	1.560009	0.126523	0.29522	-4.69586	0.416916	0.661867	0.926614	-0.15848	0.351733	0.615532	0.448848	0.012333	0.086329	0.29037	0.097911	0.228459
Cyanobacteria	1.745289	2.36204	2.019554	0.050067	0.175234	-3.95085	1.128192	0.333556	0.583723	0.214459	0.728666	0.871088	-0.48997	0.413805	0.59698	-0.27551	0.657158	0.676387
Deferribacteres	0.199303	0.436928	0.85656	0.396718	0.555405	-5.19121	3.664427	0.034435	0.241048	0.587654	0.004713	0.032992	-0.22539	0.186768	0.435792	0.362261	0.080504	0.228459
Firmicutes	-0.30705	14.74676	-1.24242	0.221213	0.387122	-5.08924	0.068914	0.933516	0.933516	-0.45294	0.012494	0.043729	0.346845	0.056268	0.196937	-0.1061	0.551198	0.676387
Proteobacteria	-0.27574	7.159348	-0.67783	0.501726	0.585346	-5.5809	0.194471	0.824032	0.933516	-0.32295	0.26414	0.615532	-0.22191	0.44938	0.59698	-0.54486	0.068004	0.228459
Tenericutes	0.160863	6.166856	0.357134	0.722842	0.722842	-5.73426	1.587194	0.21693	0.583723	-0.0781	0.805918	0.871088	-0.05718	0.859797	0.859797	-0.13528	0.676387	0.676387

OTUs without vendor × strain interactions at 24 weeks			Main Effects - Vendor (Jax relative to HSD)						Main Effects - Strain			A/J relative to BALB/c			C57BL/6 relative to A/J			C57BL/6 relative to BALB/c		
**Phylum**	**Family**	**Operational taxonomic unit (OTU)**	**logFC**	**AveExpr**	**t**	**P.Value**	**adj.P.Val**	**B**	**F**	**P.Value**	**adj.P.Val**	**logFC**	**P.Value**	**adj.P.Val**	**logFC**	**P.Value**	**adj.P.Val**	**logFC**	**P.Value**	**adj.P.Val**
No BLAST hit		No BLAST hit	-0.82242	1.064069	-1.77664	0.083199	0.141438	-5.08319	7.232697	0.002075	0.017641	-0.23627	0.547441	0.750924	0.536924	0.202647	0.522528	0.300657	0.381077	0.699971
Actinobacteria	*Coriobacteriaceae*	family *Coriobacteriaceae* , unidentified species	-1.26066	0.427705	-4.04371	0.000232	0.000563	0.348678	3.402456	0.043143	0.091679	-0.19013	0.486198	0.718727	-0.11622	0.685345	0.843994	-0.30634	0.196131	0.583694
Bacteroidetes	[*Odoribacteraceae*]	*Odoribacter* sp.	-5.89398	3.110877	-6.21135	2.34E-07	1.59E-06	6.926414	3.830097	0.030027	0.072924	-0.93697	0.163491	0.326981	1.129098	0.130972	0.494784	0.192123	0.746427	0.793079
Bacteroidetes	*Bacteroidaceae*	*Bacteroides uniformis*	-3.28182	2.260267	-6.07587	3.64E-07	1.60E-06	6.506257	2.148681	0.129851	0.167178	0.188528	0.66258	0.750924	-0.1523	0.734575	0.861225	0.036223	0.921319	0.921319
Bacteroidetes	*Bacteroidaceae*	*Bacteroides* sp.	-7.40371	5.064551	-15.4197	1.80E-18	3.06E-17	31.53782	3.155229	0.05336	0.104899	0.334427	0.390551	0.632321	0.014796	0.972365	0.975519	0.349224	0.3178	0.699971
Bacteroidetes	*Porphyromonadaceae*	*Parabacteroides* sp.	-3.59314	3.438668	-5.53808	2.08E-06	7.06E-06	4.698022	4.921833	0.01225	0.041648	-0.25557	0.637794	0.750924	0.667118	0.245895	0.522528	0.411547	0.382015	0.699971
Bacteroidetes	*Prevotellaceae*	*Prevotella* sp.	-5.37319	3.428075	-9.62416	5.50E-12	6.23E-11	17.09321	5.668112	0.006786	0.02884	-0.32428	0.435778	0.673475	0.512085	0.233059	0.522528	0.18781	0.591711	0.768364
Bacteroidetes	*Rikenellaceae*	AF12 sp.	-8.4805	4.606477	-19.7895	2.50E-22	8.52E-21	39.53086	6.424325	0.003794	0.01843	0.521304	0.126573	0.307391	-0.55421	0.105801	0.449654	-0.03291	0.906697	0.921319
Bacteroidetes	*Rikenellaceae*	family *Rikenellaceae* , unidentified species	-2.20516	9.530031	-1.13733	0.262138	0.371362	-6.29712	0.284853	0.753631	0.776468	1.559537	0.31501	0.563702	-2.12008	0.195612	0.522528	-0.56054	0.675822	0.772664
Bacteroidetes	S24-7	family 24-7, unidentified species	-0.44439	13.51689	-1.08629	0.283824	0.386001	-6.35301	5.328259	0.008861	0.033475	-1.36796	0.000144	0.004911	1.484913	9.45E-05	0.003213	0.116956	0.681762	0.772664
Cyanobacteria	*Streptophyta*	order *Streptophyta* , unidentified species	0.877652	1.486693	1.603274	0.116707	0.188954	-5.48765	2.685034	0.080448	0.136761	1.327039	0.022724	0.085845	-0.71358	0.225359	0.522528	0.613461	0.202502	0.583694
Cyanobacteria		order YS2, unidentified species	-0.70629	0.915329	-1.16912	0.249246	0.368451	-5.70647	17.7281	3.03E-06	0.000103	-1.41617	0.001045	0.017762	1.57833	0.00073	0.012404	0.162156	0.648411	0.772664
Deferribacteres	*Deferribacteraceae*	*Mucispirillum schaedleri*	-4.67338	1.783049	-5.36662	3.61E-06	1.12E-05	4.249139	10.15678	0.000269	0.00305	1.542762	0.047844	0.153519	-0.49622	0.532034	0.786485	1.046542	0.111795	0.568116
Firmicutes	[*Mogibacteriaceae*]	family [*Mogibacteriaceae*], unidentified species	0.616318	4.438277	0.985128	0.330456	0.408957	-6.41617	0.032051	0.968482	0.968482	-0.22159	0.648063	0.750924	-0.47798	0.345647	0.618527	-0.69957	0.099621	0.568116
Firmicutes	*Clostridiaceae*	Candidatus *Arthromitus*	-1.46503	0.441103	-2.94394	0.005364	0.010728	-2.47089	2.32487	0.110814	0.163839	0.189227	0.603145	0.750924	0.23226	0.561914	0.796046	0.421486	0.201184	0.583694
Firmicutes	*Clostridiaceae*	*Clostridium* sp.	1.534854	3.277779	1.453714	0.153792	0.237679	-5.66578	0.865561	0.428513	0.485648	-1.3817	0.049668	0.153519	0.936574	0.189375	0.522528	-0.44513	0.452922	0.699971
Firmicutes		order *Clostridiales* , unidentified species	-0.21011	13.44579	-0.57016	0.571743	0.627073	-6.77909	4.524454	0.016899	0.052232	0.710298	0.020446	0.085845	-0.52977	0.094542	0.449654	0.180524	0.484058	0.715564
Firmicutes	*Dehalobacteriaceae*	*Dehalobacterium* sp.	-0.55498	3.926716	-0.97216	0.336788	0.408957	-6.46921	3.109061	0.055535	0.104899	1.285455	0.007084	0.051235	-0.95808	0.050971	0.433257	0.327378	0.41025	0.699971
Firmicutes	*Erysipelotrichaceae*	*Coprobacillus* sp.	0.125797	3.736245	0.122919	0.902784	0.930141	-6.90669	0.310525	0.734806	0.776468	-1.18598	0.142039	0.310841	-0.94791	0.285948	0.555077	-2.13389	0.004629	0.157371
Firmicutes	*Erysipelotrichaceae*	family *Erysipelotrichaceae* , unidentified species	-0.83215	2.570665	-0.98652	0.329784	0.408957	-6.37181	2.512163	0.093755	0.151793	-0.86554	0.201931	0.381426	0.111362	0.876787	0.961637	-0.75417	0.20601	0.583694
Firmicutes	*Lachnospiraceae*	*Blautia* sp.	-0.28207	0.840598	-0.74326	0.461649	0.523202	-6.48255	4.273518	0.020761	0.054315	0.620524	0.080919	0.229271	0.068285	0.845755	0.958522	0.688808	0.024682	0.419599
Firmicutes	*Lachnospiraceae*	*Coprococcus* sp.	-0.02764	6.479742	-0.04214	0.966597	0.966597	-6.94258	1.212582	0.308079	0.374096	0.25794	0.562231	0.750924	0.24	0.663054	0.843994	0.698398	0.12976	0.568116
Firmicutes	*Lachnospiraceae*	*Ruminococcus gnavus*	-1.30551	7.220752	-2.34862	0.023849	0.042677	-4.32053	2.130859	0.131959	0.167178	0.458399	0.383432	0.632321	-0.66803	0.157581	0.522528	-0.41009	0.290875	0.699971
Firmicutes	*Lactobacillaceae*	*Lactobacillus* sp.	-2.52218	4.233453	-3.02392	0.004333	0.009209	-2.75652	6.761474	0.002944	0.01843	1.959961	0.007535	0.051235	-1.47269	0.065327	0.436319	0.487266	0.444176	0.699971
Firmicutes	*Peptococcaceae*	family *Peptococcaceae* , unidentified species	-4.19301	1.729033	-7.38659	5.32E-09	4.52E-08	10.55307	11.10153	0.000145	0.002467	1.116157	0.019987	0.085845	-0.9773	0.045382	0.433257	0.138853	0.722698	0.792636
Firmicutes	*Ruminococcaceae*	family *Ruminococcaceae* , unidentified species	0.156286	10.40623	0.340963	0.734908	0.78084	-6.88454	6.471646	0.003661	0.01843	1.191777	0.002214	0.025095	-0.69575	0.076997	0.436319	0.496029	0.125057	0.568116
Proteobacteria	*Alcaligenaceae*	*Sutterella* sp.	-5.32686	4.228384	-5.97375	5.06E-07	1.91E-06	6.162252	3.421316	0.042453	0.091679	-0.18166	0.77325	0.821578	-0.46891	0.508697	0.786168	-0.65057	0.255284	0.667667
Proteobacteria	*Desulfovibrionaceae*	*Bilophila* sp.	-5.56889	2.357437	-6.06589	3.76E-07	1.60E-06	6.467249	2.709339	0.078742	0.136761	0.372117	0.574772	0.750924	-0.07557	0.916823	0.974124	0.29655	0.610172	0.768364
Proteobacteria	*Desulfovibrionaceae*	*Desulfovibrio* C21_c20	-4.1745	1.109276	-6.08686	3.51E-07	1.60E-06	6.539271	2.23782	0.119824	0.167178	-0.148	0.801741	0.826037	-0.25991	0.679213	0.843994	-0.40791	0.423344	0.699971
Proteobacteria	*Desulfovibrionaceae*	*Desulfovibrio* sp.	-2.86734	1.050284	-3.2965	0.002053	0.004653	-1.79183	1.009838	0.373336	0.437704	-0.09109	0.915921	0.915921	-0.34603	0.695054	0.843994	-0.43712	0.548053	0.768364
Proteobacteria	*Halomonadaceae*	*Halomonas* sp.	-0.62543	0.283442	-2.6431	0.011656	0.022017	-3.18543	2.324679	0.110833	0.163839	-0.59242	0.017534	0.085845	0.274502	0.293864	0.555077	-0.31792	0.133674	0.568116
Proteobacteria	*mitochondria*	*Zea luxurians*	0.485274	2.173034	0.835067	0.408618	0.47907	-6.55468	2.124181	0.132759	0.167178	0.89139	0.098634	0.257965	0.016634	0.975519	0.975519	0.908025	0.049284	0.558551
Tenericutes	*Anaeroplasmataceae*	*Anaeroplasma* sp.	5.557336	8.14647	5.256845	5.15E-06	1.46E-05	3.704661	0.750576	0.478598	0.524914	-0.26891	0.749536	0.821578	0.676986	0.445822	0.734409	0.408077	0.577288	0.768364
TM7	F16	family F16, unidentified species	-3.27032	1.201684	-4.0582	0.000222	0.000563	0.379608	4.273166	0.020767	0.054315	0.933763	0.146278	0.310841	-0.51355	0.453605	0.734409	0.420213	0.448646	0.699971

Table S8
